# The wheat NLR pair RXL/Pm5e confers resistance to powdery mildew

**DOI:** 10.1111/pbi.14584

**Published:** 2025-01-22

**Authors:** Guanghao Guo, Kaihong Bai, Yikun Hou, Zhen Gong, Huaizhi Zhang, Qiuhong Wu, Ping Lu, Miaomiao Li, Lingli Dong, Jingzhong Xie, Yongxing Chen, Panpan Zhang, Keyu Zhu, Beibei Li, Wenling Li, Lei Dong, Yijun Yang, Dan Qiu, Gaojie Wang, Hee‐Kyung Ahn, He Zhao, Chengguo Yuan, Wenqi Shi, Minfeng Xue, Lijun Yang, Dazao Yu, Yusheng Zhao, Yuhang Chen, Hongjie Li, Tiezhu Hu, Guan‐Zhu Han, Jonathan D G Jones, Zhiyong Liu

**Affiliations:** ^1^ Key Laboratory of Seed Innovation, Institute of Genetics and Developmental Biology Chinese Academy of Sciences Beijing China; ^2^ The Sainsbury Laboratory University of East Anglia Norwich UK; ^3^ School of Life Sciences Zhengzhou University Zhengzhou China; ^4^ College of Advanced Agricultural Sciences University of Chinese Academy of Sciences Beijing China; ^5^ College of Life Sciences Nanjing Normal University Nanjing Jiangsu China; ^6^ Institute of Biotechnology Xianghu Laboratory Hangzhou Zhejiang China; ^7^ Tea Research Institute Yunnan Academy of Agricultural Sciences Kunming Yunnan China; ^8^ Hebei Gaoyi Stock Seed Farm Gaoyi Hebei China; ^9^ Institute of Plant Protection and Soil Science Hubei Academy of Agricultural Sciences Wuhan China; ^10^ Henan Institute of Science and Technology Xinxiang Henan Province China; ^11^ Hainan Seed Industry Laboratory Sanya City Hainan Province China; ^12^ Present address: Institute of Molecular Plant Sciences, School of Biological Sciences University of Edinburgh Edinburgh United Kingdom

**Keywords:** wheat, powdery mildew, NLR pair, hetero‐complexes, resistance mechanism

## Abstract

Powdery mildew poses a significant threat to global wheat production and most cloned and deployed resistance genes for wheat breeding encode nucleotide‐binding and leucine‐rich repeat (NLR) immune receptors. Although two genetically linked NLRs function together as an NLR pair have been reported in other species, this phenomenon has been relatively less studied in wheat. Here, we demonstrate that two tightly linked NLR genes, *RXL* and *Pm5e*, arranged in a head‐to‐head orientation, function together as an *NLR* pair to mediate powdery mildew resistance in wheat. The resistance function of the *RXL*/*Pm5e* pair is validated by mutagenesis, gene silencing, and gene‐editing assays. Interestingly, both *RXL* and *Pm5e* encode atypical NLRs, with RXL possessing a truncated NB‐ARC (nucleotide binding adaptor shared by APAF‐1, plant R proteins and CED‐4) domain and Pm5e featuring an atypical coiled‐coil (CC) domain. Notably, RXL and Pm5e lack an integrated domain associated with effector recognition found in all previously reported NLR pairs. Additionally, RXL and Pm5e exhibit a preference for forming hetero‐complexes rather than homo‐complexes, highlighting their cooperative role in disease resistance. We further show that the CC domain of Pm5e specifically suppresses the hypersensitive response induced by the CC domain of RXL through competitive interaction, revealing regulatory mechanisms within this NLR pair. Our study sheds light on the molecular mechanism underlying *RXL*/*Pm5e‐*mediated powdery mildew resistance and provides a new example of an *NLR* pair in wheat disease resistance.

## Introduction

Wheat (*Triticum aestivum*) is a critical staple crop that supports the sustenance of more than one‐third of the global population. Wheat powdery mildew caused by *Blumeria graminis* f. sp. *tritici* (*Bgt*) is an epidemic disease and leads to around 10%–40% of total yield losses (Savary *et al*., 2019). Host resistance plays a crucial role in disease control, serving as a key strategy for reducing pesticide dependence in agriculture (Sánchez‐Martín *et al*., 2021). International efforts towards genetic resistance breeding to control powdery mildew and numerous resistance (*R*) genes effective against wheat powdery mildew (*Pm* genes) have been genetically defined and strategically deployed to combat this destructive disease (Dracatos *et al*., 2023). As of now, 21 wheat *Pm* genes have been successfully cloned through various approaches, including map‐based cloning (*Pm3*, *Pm5*, *Pm13*, *Pm21*, *Pm24*, *Pm36*, *Pm38*, *Pm41*, *Pm46*, *Pm55*, *Pm57*, *Pm60*, *Pm69* and *PmTR1*/*PmTR3*) (Han *et al*., 2024; He *et al*., 2018; Krattinger *et al*., 2009; Li *et al*., 2020, 2024; Lu *et al*., 2020, 2024; Moore *et al*., 2015; Xie *et al*., 2020; Yahiaoui *et al*., 2004; Zhao *et al*., 2024) and target sequence capture approaches, including MutChromSeq (*Pm2* and *Pm4*) (Sanchez‐Martin *et al*., 2016; Sánchez‐Martín *et al*., 2021) and MutRenSeq (*Pm1* and *Pm21*) (Hewitt *et al*., 2021; Xing *et al*., 2018). Furthermore, association mapping (*WTK4*) (Gaurav *et al*., 2022) and homology‐based cloning (*Pm8*, *Pm12*, and *Pm17*) (Hurni *et al*., 2013; Singh *et al*., 2018; Zhu *et al*., 2022) have also proven useful. *Pm38* and *Pm46* are pleiotropic resistance genes that confer adult resistance not only to powdery mildew but also to wheat leaf rust, strip rust and stem rust. *Pm38* encodes a putative ABC transporter (Krattinger *et al*., 2009), while *Pm46* encodes a hexose transporter (Moore *et al*., 2015). *Pm24*, *Pm36*, *Pm57* and *WTK4* encode wheat‐tandem kinase proteins (Gaurav *et al*., 2022; Li *et al*., 2024; Lu *et al*., 2020; Zhao *et al*., 2024), *Pm13* encodes a fusion protein with mixed lineage kinase domain‐like (MLKL) and serine/threonine kinase (STK) domains (Li *et al*., 2024), and *Pm4* encodes a putative chimeric protein of a STK and multiple C2 domains and transmembrane regions (Sánchez‐Martín *et al*., 2021). The other 13 *Pm* genes encode nucleotide‐binding and leucine‐rich repeat (NLR) receptors with N‐terminal coiled‐coil (CC) domains, commonly referred to as CNLs (Coiled‐coil NLRs).

NLRs play pivotal roles in plant immunity. The C‐terminal leucine‐rich repeat (LRR) domain recognizes specific pathogen avirulence (AVR) effector proteins, either directly or indirectly, inducing a conformational change in NLRs that facilitates oligomerization (Ma *et al*., 2020; Martin *et al*., 2020; Wang *et al*., 2019). The NB‐ARC (nucleotide binding adaptor shared by APAF‐1, plant R proteins, and CED‐4) domain serves as a molecular switch, regulating NLR activation by binding ADP or ATP and NB‐ARC domains consist of a nucleotide binding domain (NBD), a helical domain (HD1) and a winged helix domain (WHD) (Wang *et al*., 2019). Self‐association of CC domains (including MLA10, Sr33, Sr50, Pm21 and ZAR1) can trigger localized cell death termed hypersensitive response (HR) in the absence of pathogens, which supports the idea that oligomerization is crucial for plant NLR activation and signalling (Bai *et al*., 2012; Bernoux *et al*., 2011; Cesari *et al*., 2016; Gao *et al*., 2020; Jacob *et al*., 2021; Krasileva *et al*., 2010). Upon activation, CNLs ZAR1 in *Arabidopsis* and Sr35 in wheat can form pentameric wheel‐like complexes, known as resistosomes that insert into the plasma membrane, acting as calcium‐permeable channels through CC domains (Bi *et al*., 2021; Förderer *et al*., 2022).

In most instances, individual NLRs are capable of simultaneous recognition of effectors and initiation of immunity (Cesari *et al*., 2014; Jones and Dangl, 2006). Nevertheless, several NLRs have been reported to function in pairs, where the sensor NLR detects AVRs through an integrated domain (ID), and the genetically linked executor NLR, which also known as helper NLR, is responsible for initiating the immune response (Cesari *et al*., 2013, 2014; De la Concepcion *et al*., 2018; Le Roux *et al*., 2015; Maqbool *et al*., 2015; Sarris *et al*., 2015). The genes encoding these corresponding NLR pairs exhibit a consistently conserved organizational pattern, characterized by close linkage and arranged in inverted orientation. Both paired rice immune receptors RGA4/RGA5 and Pik‐1/Pik‐2 can recognize effectors from *Magnaporthe oryzae* by direct binding with an integrated heavy metal‐associated (HMA) domain (De la Concepcion *et al*., 2018; Maqbool *et al*., 2015). In the *Arabidopsis* RPS4/RRS1 pair, sensor NLR RRS1‐R is able to recognize directly AvrRPS4 from *Pseudomonas syringae* pv. *pisi* and PopP2 from *Ralstonia solanacearum* directly via a C‐terminal integrated WRKY domain (Guo *et al*., 2020; Le Roux *et al*., 2015; Sarris *et al*., 2015). In wheat, while both Lr10 and RGA2 have been reported as essential for leaf rust resistance, the relationship between them remains unclear (Loutre *et al*., 2009).

We previously described the powdery mildew resistance gene *Pm5e* (MK955156), which encodes a CNL protein and an amino acid located in the C‐terminal region is crucial for its functionality (Xie *et al*., 2020). Intriguingly, another CNL gene *RXL* (*Rx‐CC‐like*) is arranged head‐to‐head with *Pm5e* in the mapping region. When *Pm5e* is introduced alone into susceptible Fielder plants, transgenic plants containing *Pm5e* confer high resistance to powdery mildew. In contrast, transgenic plants expressing *RXL* alone do not exhibit such resistance and *RXL* in Chinese Spring (*RXL‐CS, TraesCS7B03G1192600*), which is susceptible to powdery mildew, is the same as *RXL*. Based on this evidence, we previously believed that *Pm5e* alone could function effectively (Xie *et al*., 2020). However, to our surprise, four ethyl methane sulfonate (EMS)‐susceptible mutants were discovered to contain non‐synonymous mutations in the *RXL*, while *Pm5e* remained unaltered (Xie *et al*., 2020), prompting us to investigate whether *RXL* and *Pm5e* function together as an *NLR* pair.

Here, we reveal that *RXL* and *Pm5e*, which are tightly linked and in a head‐to‐head orientation in the genome, function as an *NLR* pair to mediate powdery mildew resistance in wheat. Significantly, *RXL* and *Pm5e* encode atypical CNL proteins that predominantly form heterocomplexes rather than homocomplexes, highlighting their collaborative role in bolstering disease resistance mechanisms. We further show that the CC domain of Pm5e specifically suppresses the hypersensitive response induced by the CC domain of RXL through competitive interaction, revealing regulatory mechanisms within this NLR pair.

## Results

### The digenic locus containing *RXL* and *Pm5e* confers resistance to powdery mildew

**Figure 1:**
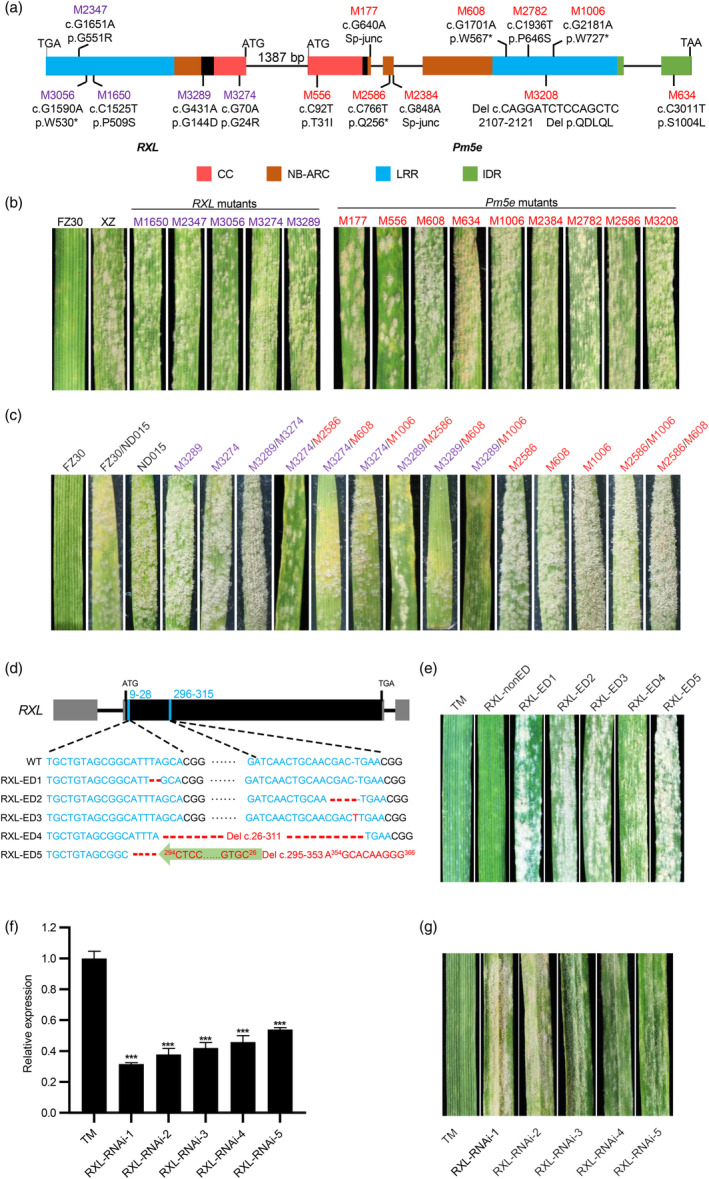
Both *RXL* and *Pm5e* are required for powdery mildew resistance. (a) The gene orders and structures of *RXL* and *Pm5e*, with mutations marked in different colours (purple for *RXL* and red for *Pm5e*) and their predicted effects on the translated protein in susceptible mutant lines. Boxes, exons; lines, introns. The domains including coiled‐coil (CC), NB‐ARC (nucleotide binding adaptor shared by APAF‐1, plant R proteins, and CED‐4), leucine‐rich repeat (LRR) and intrinsic disorder region (IDR) are highlighted in red, brown, blue and green, respectively. The linkers connecting the CC and NB‐ARC domains are highlighted in black. (b) Infection phenotypes of the *RXL* (purple) and *Pm5e* (red) susceptible mutants inoculated with *Blumeria graminis* f. sp. *tritici* (*Bgt*) isolate E20 at 14 days post‐inoculation. (c) Macroscopic infection phenotypes of representative detached wheat leaves from resistant cultivar (FZ30), susceptible cultivar (ND015), susceptible mutants of *RXL* (purple) and *Pm5e* (red) and their different F_1_ hybrids after inoculation. (d) Edited coding sequences of *RXL* gene editing (RXL‐ED) mutants. The blue line signifies the guide RNA site, and sequence modifications in the gene editing mutants are highlighted in red. (e) Macroscopic infection phenotypes of RXL‐ED mutants (RXL‐ED1 to RXL‐ED5) after *Bgt* inoculation. *RXL* non‐edited plants (RXL‐nonED) was used as control. (f) The gene expression of *RXL* in *RXL* RNAi lines. (g) Infection phenotypes of *RXL* RNAi lines after *Bgt* inoculation. The relative gene expression was standardized to the expression of *RXL* in TM (expression = 1). *TaACTIN* was used as an internal control. Error bars represent the standard error of the means (SEMs) of three independent experiments. Statistically significant differences (Student's *t*‐test): ***P < 0.001.

*RXL* and *Pm5e* are in a head‐to‐head orientation with only 1387 bp between their start codons (Figure 1a), suggesting that they may be co‐regulated at the transcript level. Upon *Bgt* inoculation, a significant upregulation of both *RXL* and *Pm5e* were observed, suggesting their co‐regulation (Figure S1). To investigate the potential function of *RXL* and *Pm5e* in conferring resistance to powdery mildew as an *NLR* pair, we first conducted screenings on EMS mutagenized Fuzhuang 30 (FZ30) and Tangmai 4 (TM), both carrying functional *RXL* and *Pm5e*, to identify susceptible mutants. Finally, we identified five independent *RXL* mutants from FZ30 and nine independent *Pm5e* mutants, including three from TM (M556, M608 and M2782) and six from FZ30, which include previously characterized mutants (Xie *et al*., 2020). All these mutants contain non‐synonymous mutations in the *RXL* and *Pm5e* genes, respectively (Figure 1a,b). According to sequence amplification, *Pm5e* is not mutated in all five *RXL* mutants, and *RXL* is not mutated in all nine *Pm5e* mutants. To validate the causal relationship between loss of resistance and mutations in the *RXL* or *Pm5e*, we selected independent loss‐of‐function mutants homozygous for *RXL* (M1650, M3274 and M3289) and *Pm5e* (M608 and M1006), respectively, to make crosses with their corresponding wild types (FZ30 or TM) to develop segregating populations. All the *F*_2:3_ progenies from the five populations segregated in a 1:2:1 ratio for homozygous resistant, segregating and homozygous susceptible (Table S1). The mutation site‐designed SNP markers from the *RXL* or *Pm5e* genes exhibited co‐segregation with the infection type in respective populations (Figure S2), indicating that both *RXL* and *Pm5e* are necessary for resistance to powdery mildew.

To further validate the genetic interaction of *RXL* and *Pm5e* for powdery mildew resistance, we selected two *RXL* loss‐of‐function mutants (M3274 and M3289) to make crosses with three *Pm5e* loss‐of‐function mutants (M608, M1006 and M2586). As controls, we also carried out crosses between different *RXL* mutants as well as between different *Pm5e* mutants. As expected, a significantly increased level of resistance was observed in the *F*_1_ hybrids resulting from crosses between *RXL* and *Pm5e* mutants (Figure 1c), akin to the phenotypes observed in the F_1_ plants from crosses of the resistant parent FZ30 and the susceptible parent ND015, which was utilized to establish the population for mapping and cloning *Pm5e* (Xie *et al*., 2020). Conversely, the *F*_1_ hybrids resulting from crosses of *RXL* mutants or *Pm5e* mutants themselves exhibited high susceptibility to *Bgt* E20, similar to the parent mutant lines (Figure 1c). These results imply that *RXL* and *Pm5e* function interdependently.

We then performed barley strip mosaic virus‐induced gene silencing (BSMV‐VIGS) by designing two constructs to selectively silence expression of either *RXL* or *Pm5e* in TM (Figure S3a, b). The expression of *RXL* or *Pm5e* in TM leaves infected with BSMV: *RXL* or BSMV: *Pm5e* decreased significantly compared with TM plants infected with control BSMV:γ (Figure S3c, d). As a result, sporulating mildew colonies observed on the leaves were consistent with reduced *RXL* or *Pm5e* expression level, while the resistant phenotype was maintained on leaves infected with control vector (Figure S3e). The BSMV‐VIGS results further confirm that maintaining robust powdery mildew resistance requires sufficient expression of both *RXL* and *Pm5e*.

To further test whether the loss of *RXL* function is sufficient to negate resistance to wheat powdery mildew, we developed *RXL* knockout plants in the TM/Fielder *F*_1_ background using CRISPR/Cas9 technology. The reason for using this background is to enhance genetic manipulation efficiency, as TM is an uncommon transgenic cultivar. Out of 13 transgenic positive T_0_ plants, we identified five *RXL* editing events. The T_0_ plants with *RXL* editing were then advanced to T_1_ generation to select plants with a homozygous *Pm5e* genotype, indicating the presence of homozygous functional *Pm5e*, for further analysis. In the T_1_ generation of five *RXL* knockout mutants with homozygous functional *Pm5e*, we detected various deletions and insertions leading to reading frames shifting and premature termination in the *RXL* gene (Figure 1d). Upon inoculation with *Bgt* E20, all five *RXL* knockout mutants (RXL‐ED1 to RXL‐ED5) exhibited severe susceptibility, while the *RXL* non‐edited plants (RXL‐nonED) remained resistant, similar to wild‐type TM (Figure 1e). Given the head‐to‐head arrangement of *RXL* and *Pm5e*, there is a chance that the guide RNA target region is situated on the *Pm5e* promoter, which might affect the expression of both genes. To exclude this possibility, we examined the expression of *RXL* and *Pm5e* to ascertain any potential impact on their expression in these gene‐edited mutants. There was no significant difference in the expression of *RXL* and *Pm5e* between the *RXL*‐edited mutants and the non‐edited controls (Figure S4a). These findings indicated that the absence of *RXL* function abolishes resistance to powdery mildew, even in the presence of functional *Pm5e*, implying a collaborative role of *RXL* and *Pm5e* in conferring resistance to powdery mildew.

We also implemented RNA interference (RNAi) to downregulate *RXL* expression in the heterozygous TM/Fielder F_1_ plants, and advanced the transgenic T_0_ plants to T_1_ generation. We identified five positive *RXL* RNAi transgenic plants with significantly reduced *RXL* expression in the plants with homozygous *Pm5e* genotype. We also evaluated *Pm5e* expression in two *RXL* RNAi lines and found no significant changes in its expression levels (Figure S4b). All these transgenic plants showed increased susceptibility to *Bgt* E20, suggesting specific downregulation of *RXL* expression compromised the powdery mildew resistance (Figure 1f,g).

Based on the results above, we can confidently conclude that *RXL* and *Pm5e* are both required for resistance to powdery mildew. To explain why introducing *Pm5e* alone into Fielder confers resistance (Xie *et al*., 2020), we determined the sequences of *RXL* and *Pm5e* in Fielder (*TraesFLD7B01G473700.1* and *TraesFLD7B01G473800.1*) (Sato *et al*., 2021). The results showed that Pm5‐Fielder displays 55 amino acid variances compared with Pm5e, accompanied by two deletions (Figure S5c). Furthermore, a retrotransposon is inserted within the first intron of the *Pm5‐Fielder* gene (Figure S5a), belonging to the susceptible haplotype at the *Pm5* locus (Xie *et al*., 2020). In contrast, RXL and RXL‐Fielder have only four amino acid variations, indicating *RXL‐Fielder* may represent a functional allele. The evidence that introducing *Pm5e* into Fielder confers resistance to powdery mildew indicates that *RXL‐Fielder* can function with *Pm5e* to provide this resistance (Figure S5b,f). To further validate the function of *RXL‐Fielder*, we performed BSMV‐VIGS in *Pm5e* overexpression (Pm5e‐OE) transgenic lines. The expression of *RXL* or *Pm5e* in leaves infected with BSMV:*RXL‐Fielder* or BSMV:*Pm5e* significantly decreased compared to the control BSMV:γ (Figure S5d). This reduction in *RXL‐Fielder* expression correlated with decreased resistance, similar to the positive control where *Pm5e* was silenced in transgenic plants (Figure S5e). These results confirm that *RXL‐Fielder* is functional.

### Both *RXL* and *Pm5e* encode atypical CNLs

**Figure 2:**
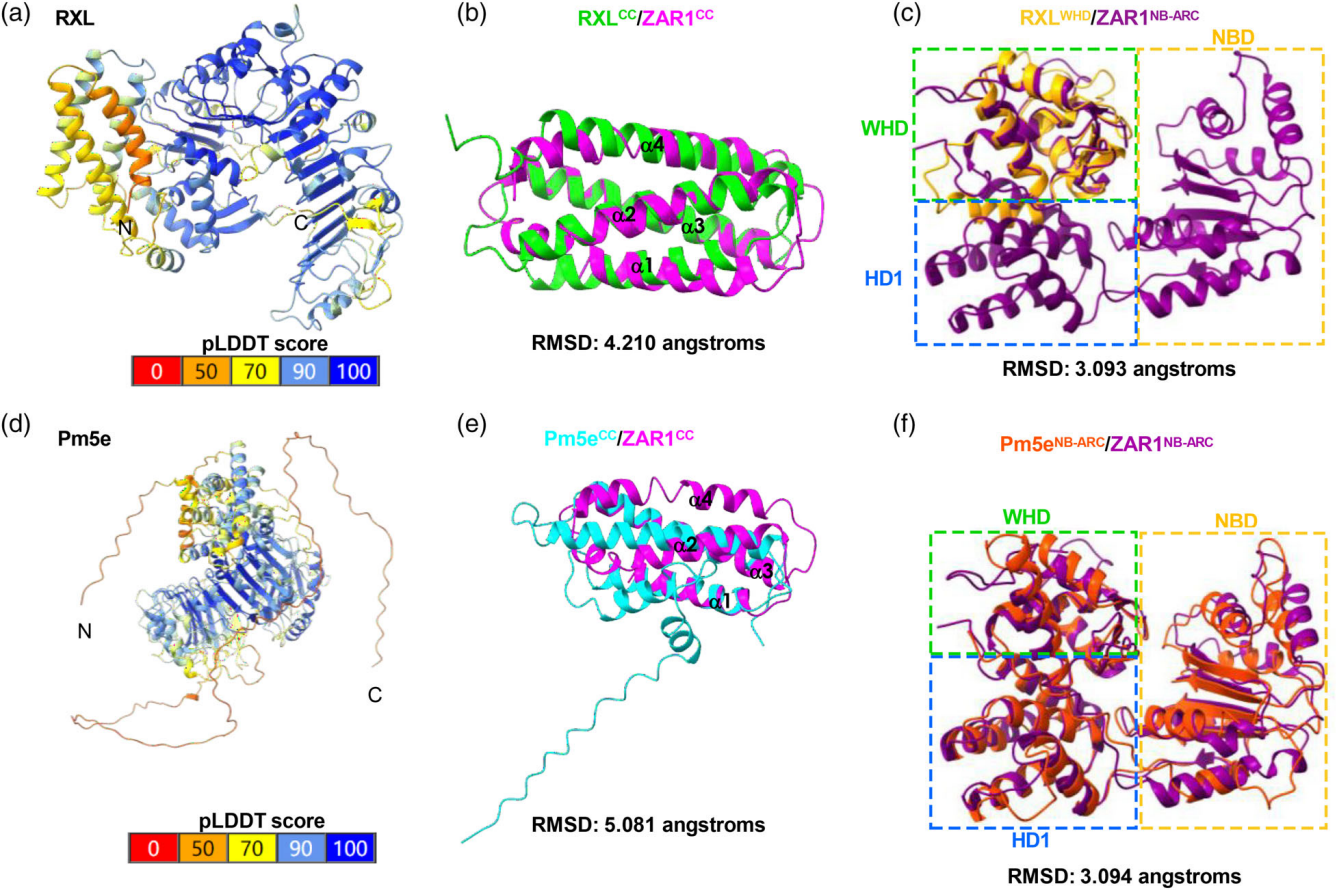
RXL and Pm5e encode atypical CNL proteins according to the protein structure prediction. (a) The RXL protein structure was predicted using AlphaFold2, and the pLDDT (predicted Local Distance Difference Test) values for the predicted full‐length protein are presented. Dark blue represents a high pLDDT value, indicating high confidence, while orange indicates a low pLDDT value, signifying low confidence. (b) Structural overlay of RXL^CC^ in lime and  ZAR1^CC^ in hot pink. (c) Structural overlay of RXL^WHD^ in orange and ZAR1^NB‐ARC^ in purple. (d) The Pm5e protein structure was predicted using AlphaFold2. pLDDT values for the predicted full‐length protein are presented. (e) Structural overlay of Pm5e^CC^ in cyan and ZAR1^CC^ in hot pink. (f) Structural overlay of Pm5e^NB‐ARC^ in orange red and ZAR1^NB‐ARC^ in purple. The RMSD (Root Mean Squared Deviation) values were shown.

AlphaFold2 (Jumper *et al*., 2021) was employed to predict the structure models of RXL and Pm5e (Figure 2a,d). The RXL model exhibits a standard CC domain (Figure 2b), a truncated NB‐ARC domain (Figure 2c), and LRR domain with 15 repeat units (Figure S6a), while the Pm5e structure model highlights an atypical CC domain (Figure 2e), followed by a canonical NB‐ARC domain (Figure 2f), and LRR domain with 17 repeat units (Figure S6b). Additionally, Pm5e includes an extended unstructured C‐terminal ‘tail’ identified as intrinsically disordered region (IDR) (Figure 2d, Figure S7). The CC domain of RXL closely resembles the CC domain of the canonical CNL ZAR1, both containing four α‐helices (Figure 2b). However, the first helix (α1) that plays a crucial role in Ca^2+^ channel formation differs markedly between the atypical Pm5e^CC^ and conventional ZAR1^CC^ (Figure 2e). Notably, while Pm5e exhibits an intact NB‐ARC domain that closely resembles ZAR1^NB‐ARC^ (Figure 2f, Figure S6b), the NB‐ARC domain of RXL comprises only the WHD segment and lacks NBD and HD1 domains, which contain essential components such as P‐loop, RNBS‐A, Kinase 2, RNBS‐B, RNBS‐C and GLPL motifs within the complete NB‐ARC domain (Figure 2c, Figure S6a). NB‐ARC domain plays a crucial role in structural remodelling during activation, contributing to the formation of the pentameric resistosomes in ZAR1 and Sr35 (Förderer *et al*., 2022; Wang *et al*., 2019). Together, these results suggest that RXL and Pm5e possibly rely upon each other for their function.

### RXL and Pm5e can form both homo‐ and hetero‐complexes, with a preference for forming hetero‐complexes

To explore whether the functional interaction between RXL and Pm5e depends on the formation of RXL and Pm5e hetero‐complexes, we first predicted the interactions between RXL and Pm5e using AlphaFold‐Multimer (AFM) (Evans *et al*., 2022). The best overall score for the RXL‐Pm5e hetero‐dimer, as predicted by AFM, was 0.86. This score is derived from a combination of a predicted Template Modelling (pTM) and the interface pTM (ipTM) score and is further supported by high pLDDT scores (Figure S8a). The AFM also predicted possible formation of RXL and Pm5e homodimers. However, the scores for RXL and Pm5e homo‐complexes are 0.43 and 0.63, respectively (Figure S8b,c). Both scores are lower than that for the RXL‐Pm5e hetero‐dimer (0.86), indicating that RXL and Pm5e are more likely to form a hetero‐complexes.

**Figure 3:**
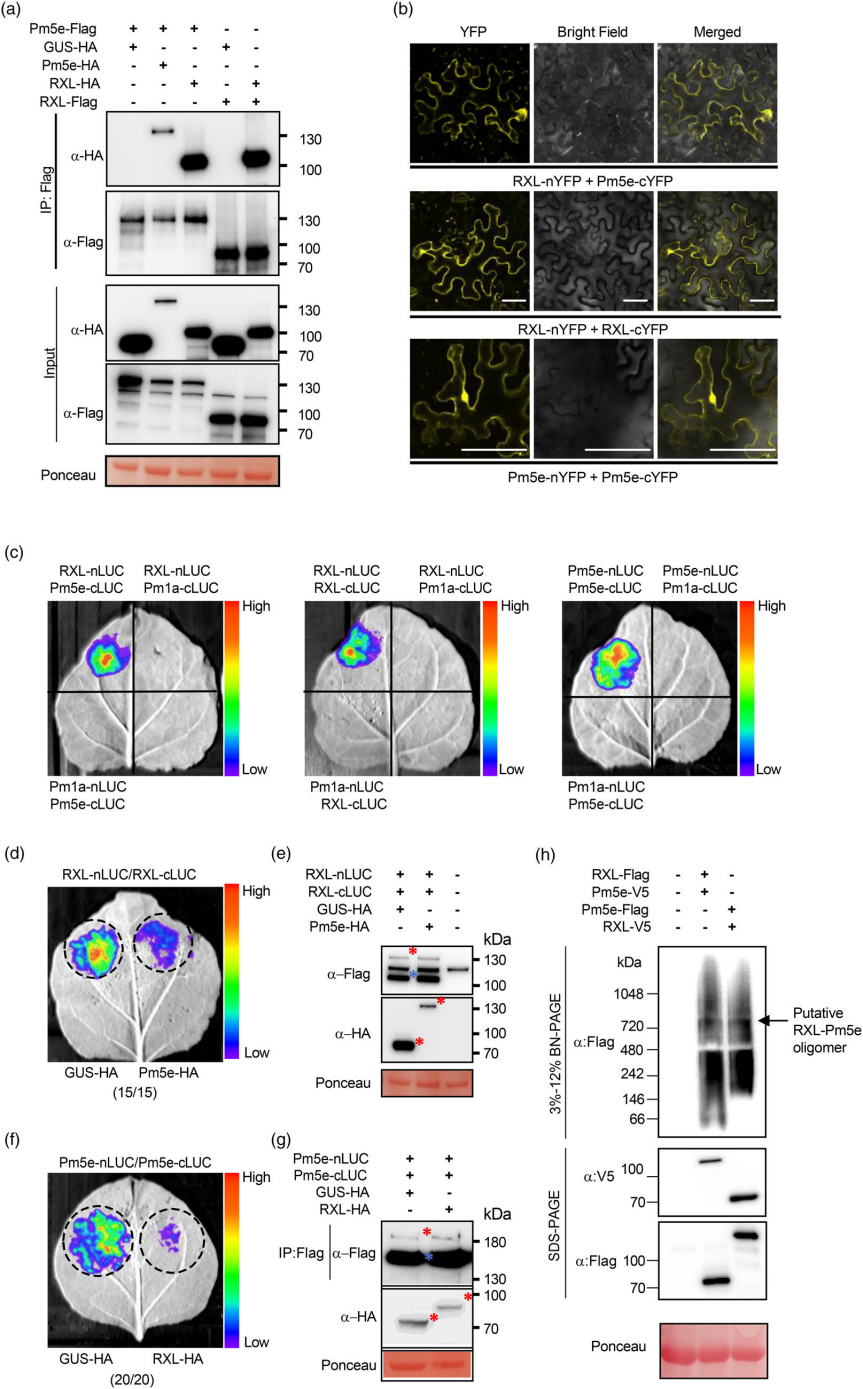
RXL and Pm5e can form both homo‐ and hetero‐complexes, with a preference for forming hetero‐complexes. (a) Identification of potential RXL and Pm5e homo‐ and heterodimeric protein interactions through co‐immunoprecipitation. RXL and Pm5e were tagged with either HA or Flag C‐terminally and were transiently expressed in *N. benthamiana*. Proteins were extracted 3 days post‐infiltration, followed by immunoblotting with anti‐HA and anti‐Flag antibodies. The presence or absence of proteins is indicated by the +/− sign (Input). Moreover, immunoprecipitations were carried out using anti‐Flag M2 beads and subsequent immunoblotting with anti‐Flag for the detection of immunoprecipitated proteins and with anti‐HA for the detection of co‐precipitated proteins (IP). Ponceau staining of the western blot membrane is shown at the bottom. (b) BiFC showed interactions between RXL and Pm5e themselves and each other. Scale bars, 50 mm. (c) Interactions between full‐length RXL and Pm5e individually as well as between each other were confirmed using split‐luciferase assays. (d, f) RXL and Pm5e prefer to form hetero‐complex were confirmed using split‐luciferase assays. The number of leaves tested and the occurrences of decreased signal are indicated in parentheses. (e, g) The abundance of fusion proteins was determined by immunoblot analysis in a transient expression assay in *N. benthamiana*. The molecular weights of the proteins are as follows: RXL‐nLUC is approximately 130 kDa, RXL‐cLUC is approximately 100 kDa, GUS‐HA is approximately 70 kDa, Pm5e‐HA is 130 kDa, Pm5e‐nLUC is approximately 180 kDa, Pm5e‐cLUC is approximately 150 kDa, and RXL‐HA is approximately 80 kDa. Due to the large size of Pm5e‐nLUC, which makes it difficult to detect, we first performed immunoprecipitation using Flag beads and then analysed the samples by western blot. The target proteins are indicated individually based on their molecular weight. (h) RXL and Pm5e can form hetero‐oligomeric complexes in *N. benthamiana*, as demonstrated by BN‐PAGE.

To experimentally validate the predicted interactions between RXL and Pm5e, we first conducted co‐immunoprecipitation (co‐IP) experiments in *Nicotiana* (*N*.) *benthamiana* plants. Possible formation of RXL and Pm5e homo‐complexes were also investigated since NLR proteins have been reported to form homo‐complexes (Cesari *et al*., 2014). Immunoblotting with anti‐Flag and anti‐HA antibodies confirmed the appropriate expression of all proteins (Figure 3a). Pm5e‐Flag specifically co‐immunoprecipitated with RXL‐HA but not with GUS (β‐glucuronidase)‐HA (Figure 3a). Interestingly, Pm5e‐HA was also co‐immunoprecipitated by Pm5e‐Flag, and RXL‐HA co‐immunoprecipitated with RXL‐Flag but not with GUS‐HA. These results suggest RXL and Pm5e may form hetero‐ and homo‐complexes. To further investigate hetero‐complex and self‐association formation between RXL and Pm5e, we performed bimolecular fluorescent complementation (BiFC) assay. The fluorescence signal resulting from the co‐expression of RXL‐nYFP and Pm5e‐cYFP was observed, indicating that RXL and Pm5e interact each other (Figure 3b). Additionally, both RXL and Pm5e also exhibited self‐association (Figure 3b). By contrast, no fluorescence signal was observed when co‐expressing RXL or Pm5e with control vectors (Figure S9a). Western blotting showed that all fusion proteins were properly expressed (Figure S9b). The heteromeric and homomeric protein interactions of RXL and Pm5e were also validated using split‐luciferase assays. Luciferase signal was detected when RXL‐nLUC and Pm5e‐cLUC were co‐expressed (Figure 3c), but not in the negative controls where RXL‐nLUC and Pm1a‐cLUC, or Pm1a‐nLUC and Pm5e‐cLUC were co‐expressed, suggesting RXL associates with Pm5e *in planta*. Additionally, luciferase signal was observed when RXL‐nLUC and RXL‐cLUC, as well as Pm5e‐nLUC and Pm5e‐cLUC, were co‐expressed, indicating self‐association of both RXL and Pm5e (Figure 3c). Western blotting showed that all fusion proteins were properly expressed (Figure S10).

To further investigate whether RXL and Pm5e predominantly form homomeric or heteromeric protein interactions, we co‐expressed HA‐tagged Pm5e with RXL‐nLUC/RXL‐cLUC and HA‐tagged RXL with Pm5e‐nLUC/Pm5e‐cLUC, using HA‐tagged GUS as a control. Compared with the control, the luciferase signals from the association of RXL‐nLUC/RXL‐cLUC decreased upon the addition of Pm5e‐HA, while the signals from Pm5e‐nLUC/Pm5e‐cLUC also decreased with the addition of RXL‐HA (Figure 3d,f). Notably, the protein expression levels of both RXL‐nLUC/RXL‐cLUC and Pm5e‐nLUC/Pm5e‐cLUC remained unchanged (Figure 3e,g). The evidence implies that RXL and Pm5e preferentially establish heteromeric complexes rather than homomeric complexes. Taken together, these results strongly suggest that RXL and Pm5e prefer to form hetero‐complexes *in planta*, in addition to self‐association capacity of RXL and Pm5e, respectively.

To further determine whether RXL and Pm5e form a hetero‐oligomeric complex *in vivo*, we performed Blue Native polyacrylamide gel electrophoresis (BN‐PAGE). Flag‐tagged RXL and Pm5e were co‐expressed with V5‐tagged Pm5e or RXL in *N. benthamiana* leaves (Figure 3h). Consistent detection of the oligomeric complex was observed using the Flag antibody to blot Flag‐tagged RXL co‐expressed with V5‐tagged Pm5e or Flag‐tagged Pm5e co‐expressed with V5‐tagged RXL (Figure 3h), suggesting the formation of hetero‐oligomeric complexes. The high‐molecular‐weight protein complexes, ranging from 800 to 1048 kDa (Figure 3h), contrasted with the predicted molecular weights of the RXL monomer (~73 kDa) and the Pm5e monomer (~119 kD). These results suggest the potential existence of RXL‐Pm5e hetero‐oligomers.

### Pm5e^CC^ specifically suppresses HR induced by RXL^CC^ through competitively interacting with RXL^CC^

**Figure 4:**
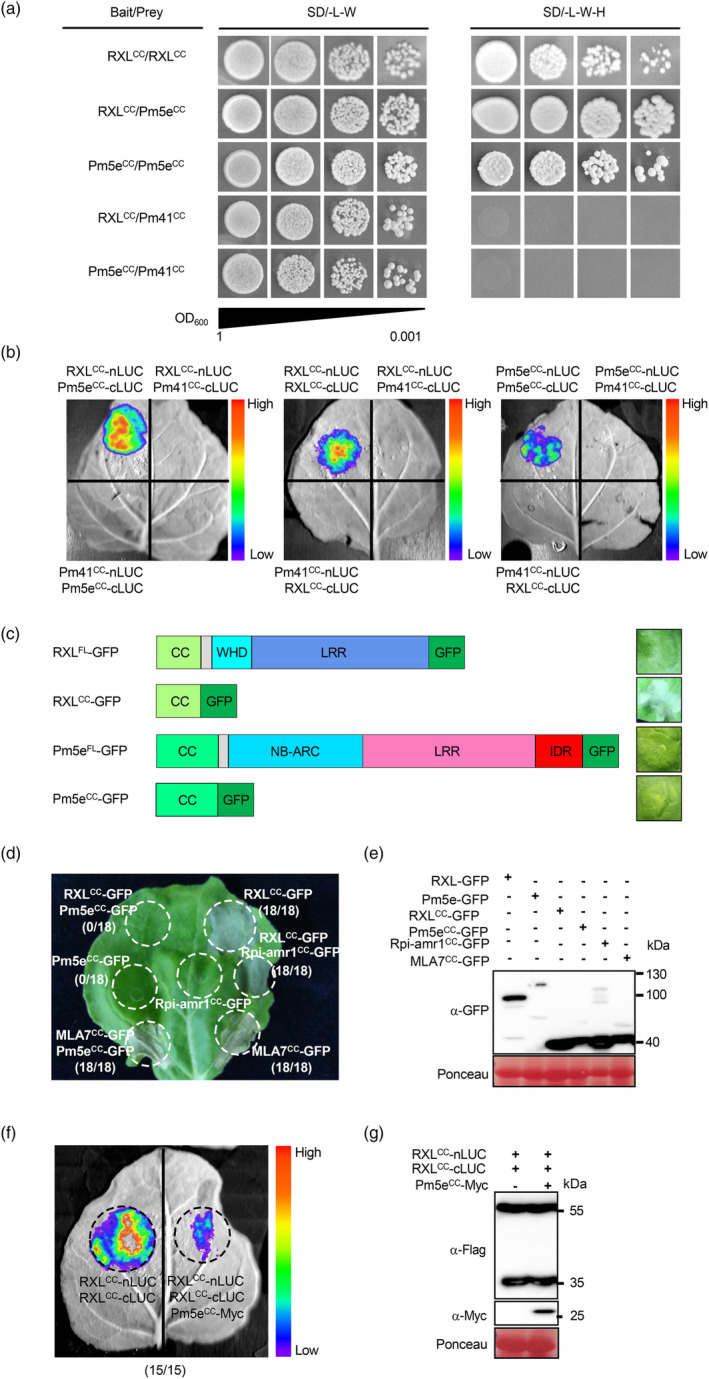
Pm5e^CC^ specifically suppresses HR induced by RXL^CC^ through competitively interacting with RXL^CC^. (a and b) Interaction between CC domains of RXL and Pm5e themselves and each other were confirmed by split‐luciferase assays and yeast two‐hybrid assays. (c) HR phenotypes of *N*. *benthamiana* leaves upon the expression of full‐length and the CC domains of RXL and Pm5e. The phenotypes were recorded 7 days after infiltration. (d) RXL^CC^‐mediated HR is suppressed by Pm5e^CC^. The constructs RXL^CC^‐GFP, Pm5e^CC^‐GFP, Rpi‐amr1^CC^‐GFP, MLA7^CC^‐GFP, RXL^CC^‐GFP and Pm5e^CC^‐GFP, RXL^CC^‐GFP and Rpi‐amr1^CC^‐GFP, MLA7^CC^‐GFP and Pm5e^CC^‐GFP were expressed in *N*. *benthamiana* and cell death was visualized 7 days after infiltration. Representative leaf phenotype of HR is shown. The number of leaves tested and occurrences of HR are indicated in parentheses. (f) Pm5e^CC^ can competitively interact with RXL^CC^. The number of leaves tested and the occurrences of decreased signal are indicated in parentheses. (e, g) The protein expression levels of fusion proteins in the *N. benthamiana* leaves were assessed through SDS‐PAGE, followed by immunoblotting with corresponding antibodies.

The CC domain has been proven to play an important role in the formation of both homo‐ and hetero‐complexes in paired NLRs (Cesari *et al*., 2014). To investigate whether the formation of RXL‐Pm5e hetero‐complexes and RXL and Pm5e homo‐complexes depends on their CC domains, the interactions between the CC domains of RXL and Pm5e were analysed using the yeast two‐hybrid system. The results showed that RXL and Pm5e CC domains interacted with themselves and on another (Figure 4a), demonstrating that RXL and Pm5e can form homo‐ and hetero‐complexes through their respective CC domains. These results were further corroborated by split‐luciferase assays (Figure 4b). Importantly, CC‐homo‐ and hetero‐interactions were specific since no interaction with the CC domain of the sequence unrelated wheat powdery mildew resistance CNL protein Pm41 (Li *et al*., 2020).

In the ZAR1 resistosome, the NB‐ARC domain of ZAR1 induces oligomerization of ZAR1 protomers and the five N‐terminal α1‐helices of ZAR1^CC^ form a solvent‐exposed structure functioning as Ca^2+^‐permeable channel to mediate immune response (Huang *et al*., 2023). Given that RXL^CC^ resembles ZAR1^CC^ (Figure 2b), whereas the α‐1 helix of Pm5e^CC^ shows significant differences compared with ZAR1^CC^ (Figure 2e), we investigated the function of RXL^CC^ and Pm5e^CC^ in conjunction with the full‐length RXL and Pm5e. All proteins were fused with C‐terminal GFP tags and were transiently overexpressed individually or together in *N. benthamiana*. The results showed that neither the full‐length RXL nor Pm5e proteins induced HR. Intriguingly, only RXL^CC^ induced robust HR, whereas Pm5e^CC^ produce no visible phenotype (Figure 4c). Interestingly, co‐expression of RXL^CC^ and Pm5e^CC^ resulted in the specific repression of HR induced by RXL^CC^, supported by the observation that Pm5e^CC^ did not affect MLA7^CC^‐induced HR and Rpi‐amr1^CC^ did not influence RXL^CC^‐induced HR (Figure 4d). Western blotting showed that all fusion proteins were properly expressed (Figure 4e). Self‐association of the CC domain has been demonstrated to induce HR in some CNLs (Bai *et al*., 2012). The self‐association of RXL^CC^ markedly decreased upon the addition of Pm5e^CC^, while protein expression of both RXL^CC^‐nLUC and RXL^CC^‐cLUC remained unaffected (Figure 4f,g). Collectively, the findings suggest that Pm5e^CC^ specifically suppresses the HR induced by RXL^CC^ through competitively interacting with RXL^CC^, indicating RXL may function as the executor NLR and Pm5e as the sensor NLR.

The conserved MHD motif in the ARC2 domain is pivotal for regulating NLR proteins. Alterations to the conserved histidine or aspartate within the MHD core sequence often result in the production of auto‐active NLR proteins, triggering a constitutive activation of immune responses and cell death (Bendahmane *et al*., 2002; Cesari *et al*., 2014). Investigation of the ARC2 domains in RXL and Pm5e revealed non‐canonical MHD motifs in both NLR proteins (Figure S11a). In RXL, the MHD motif is highly degenerated, with all three amino acids differing from the consensus MHD sequence (P_212_T_213_G_214_), resembling the RGA4 MHD motif closely (T_500_Y_501_G_502_). In Pm5e, the motif is less disturbed (V_503_H_504_H_505_) and resembles the RGA5 MHD motif (L_504_H_505_H_506_), with the central histidine conserved, flanked by two hydrophobic residues.

To determine whether mutations in the MHD motif can induce auto‐activity in RXL or Pm5e, point mutations were introduced into RXL and Pm5e and these constructs were fused with C‐terminal GFP tag. RXL_PTV_‐GFP was transiently expressed in *N. benthamiana* either alone or in combination with Pm5e‐GFP. Pm5e_VHV_‐GFP and Pm5e_VVH_‐GFP were expressed alone or co‐expressed with RXL‐GFP, respectively. Western blotting showed that all fusion proteins were properly expressed (Figure S11d). However, none of these constructs, either alone or in combination, was effective in triggering cell death (Figure S11b,c), indicating that there are some other active mechanisms in RXL/Pm5e pair.

### The RXL/Pm5e orthologs evolved from a pre‐existing head‐to‐head pair before the divergence of Triticeae

**Figure 5:**
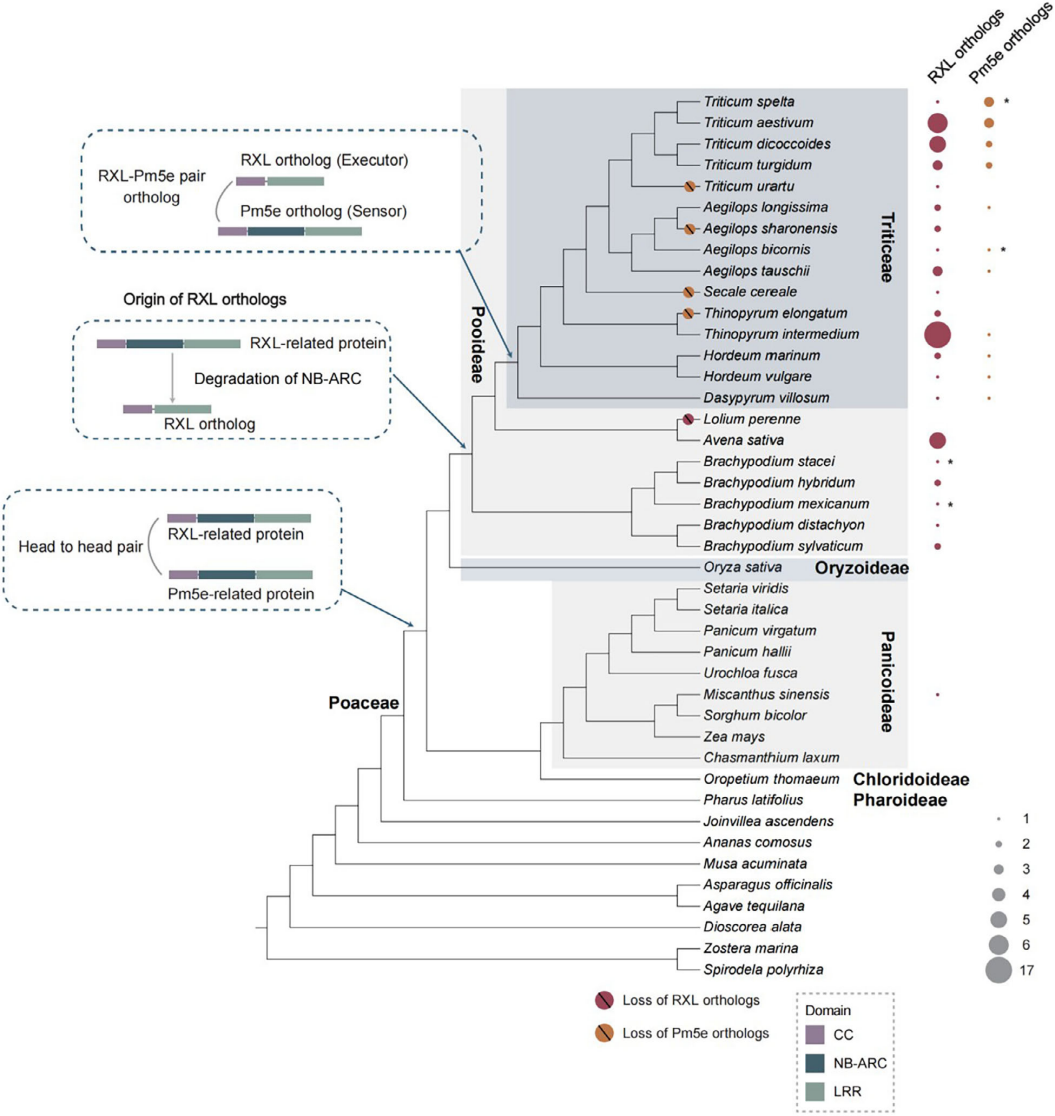
Evolutionary model of the RXL/Pm5e orthologs. The copy numbers of RXL and Pm5e orthologs for each species are shown next to the corresponding species in red and orange circles, respectively. The size of a solid circle reflects the copy number. The asterisk represents that the genes were identified through tBLASTn. The phylogeny of 42 monocots is inferred from literature (Huang *et al*., 2022) and TimeTree (https://timetree.org/). Red and orange circles with slashes on the branches indicate the loss of RXL orthologs and the loss of Pm5e orthologs, respectively. The three boxes represent the possible evolutionary scenarios of the RXL/Pm5e orthologs. First, RXL‐related NLRs and Pm5e‐related NLRs formed head‐to‐head pairs at the early evolution of Poaceae and has been maintained. Then, RXL‐related NLRs lost the NB‐ARC domain to become RXL orthologous proteins before the ancestor of Pooideae, and Pm5e orthologs originated before the last common ancestor of Triticeae. The RXL/Pm5e orthologous pair was derived from an ancestral NLR pair before the last common ancestor of Triticeae.

Through combining similarity search and phylogenetic analysis, we explored the evolutionary trajectory of the RXL/Pm5e pair orthologs across 122 representative plant species that cover the major diversity of plants, encompassing 34 species in Poaceae (Figure 5, Table S2). Phylogenetic analysis shows that Pm5e orthologs form a monophyletic group within NLRs and are distributed in 11 out of 15 Triticeae species surveyed, indicating that Pm5e orthologs might have originated at least before the last common ancestor (LCA) of Triticeae (Figure S12a, Table S3). RXL orthologs that are characterized by their CC‐LRR with broken NB‐ARC domain structure nest within the diversity of CNL proteins (UFBoot support of 99%) (Figure S12b, Table S4). RXL orthologs were identified in *Miscanthus sinensis* and all the Pooideae species surveyed (with the exception of *Lolium perenne*). However, the RXL ortholog of *M. sinensis* falls within the diversity of RXL orthologs of Pooideae, suggesting that the RXL ortholog might have derived from Pooideae through horizontal gene transfer. Together, these results suggest that RXL orthologs might have originated from an ancestral CNL via degradation of the NB‐ARC domain before the LCA of Pooideae (Figure 5). Interestingly, the number of RXL ortholog copies varies among different species, ranging from a single copy in *Hordeum vulgare* to 18 copies in *Thinopyrum intermedium*. In contrast, Pm5e orthologs typically exist as a single‐copy gene, except in some Triticeae species such as *T. aestivum* and *T. spelt* due to their polyploidy nature (Figure 5).

To further elucidate the maintenance of physical association between RXL and Pm5e, we performed phylogenetic analyses of NLR proteins from 13 representative monocot species. Our analyses reveal a long maintenance of the head‐to‐head relationship between RXL‐related and Pm5e‐related proteins. We found that the physical association between RXL and Pm5e orthologs can be traced back to an ancestral head‐to‐head pair that might have arisen in the early evolution of Poaceae, right after the divergence of Pharoideae and other Poaceae, approximately 82.5 million years ago (Mya) (Ramírez‐Barahona *et al*., 2020) (Figure S13).

## Discussion

In recent years, an increasing body of evidence has revealed plant disease resistance loci that comprise pairs of two distinct NLR proteins, rather than individual ones (Bialas *et al*., 2018). Noteworthy examples of well‐studied NLR pairs include RPS4/RRS1, RGA4/RGA5 and Pik‐1/Pik‐2, wherein a sensor NLR identifies pathogen effectors via its integrated domain (ID), and an executor NLR initiates immune signalling. In wheat, although both Lr10 and RGA2 are reported to be essential for rust resistance, the underlying resistance mechanisms are not well‐understood (Loutre *et al*., 2009). Our study provides novel insights, demonstrating that the two CNL genes, *RXL* and *Pm5e*, arranged in a head‐to‐head orientation in the genome, play a crucial and complete role in conferring resistance to powdery mildew. The indispensability of both *RXL* and *Pm5e* for disease resistance was corroborated through genetic and molecular methods.

Our understanding of NLR signalling mechanisms has been through significant expansion recently. Notably, resistosomes formed by CNL ZAR1 in *Arabidopsis* and Sr35 in wheat exhibit a strikingly conserved wheel‐like pentamer structure, functioning as Ca^2+^‐permeable influx channels. The N‐terminal α‐helix in the CC domain is crucial for ZAR1 and Sr35 resistosome function (Bi *et al*., 2021; Förderer *et al*., 2022; Wang *et al*., 2019). This suggests that the oligomeric protein complex assembly upon activation may be conserved across various plant CNLs. Furthermore, based on the structural similarity of CC domain of RXL to ZAR1 (Figure 2b), it is possible that the CC domain of RXL functions similarly to ZAR1.

In the characterized NLR pairs RPS4/RRS1 and RGA4/RGA5, the executor NLRs induce cell death exclusively, while the sensor NLRs repress the constitutive activity of the executor NLRs. Additionally, the rice NLR pair Pikp‐1/Pikp‐2 initiated immunity through receptor cooperation rather than negative regulation (Bialas *et al*., 2018). Both NLRs in these pairs possess intact TIR or CC, NB‐ARC and LRR domains. In the RXL/Pm5e pair, RXL carries a classical CC domain capable of inducing robust HR, signifying its ability to form a Ca^2+^ channel to activate immune responses. However, the α1 helix of Pm5e^CC^ differs significantly from ZAR1^CC^, suggesting an inability to form a Ca^2+^ channel and Pm5e^CC^ can specifically suppress HR induced by RXL^CC^ (Figures 2 and 4). This implies that RXL may function as an executor NLR, while Pm5e acts as a sensor NLR. Interestingly, the full‐length RXL alone fails to induce HR, despite having a similar MHD motif to RGA4, where the constitutive activity is attributed to its degenerated MHD motif (Figure 4, Figure S11). This discrepancy may be due to RXL lacking a complete NB‐ARC domain, as NBD and HD1 are absent, with only WHD remaining in the structure (Figure 2c). Pm5e possesses a fully intact NB‐ARC domain, encompassing NBD, HD1, and WHD domains, along with a MHD motif similar to RGA5 (Figure 2f, Figure S11). However, the lack of HR induction in all MHD mutants of both RXL and Pm5e suggests a distinct activation mechanism from RGA4/RGA5 (Figures 2 and 4, Figure S11). The ability of RXL^CC^ to induce HR while the full‐length RXL cannot suggests the involvement of another intramolecular interaction suppressing HR (Figure 4).

Currently, the oligomeric state and structural prerequisites for the formation of activated oligomers have yet to be reported for paired NLRs. A recent study revealed that activated pair NLR CHS3‐CSA1 alleles form distinct hetero‐oligomeric complexes (Yang *et al*., 2024). The requirement for functional CC and NB‐ARC domains for resistosome formation and channel development, respectively, suggests that RXL and Pm5e, when acting alone, may be insufficient to form a resistosome and activate immune activity. This notion leads to the hypothesis that RXL and Pm5e collaboratively form a resistosome. The evidence from BN‐PAGE, revealing the formation of a high‐molecular‐weight complex between RXL and Pm5e in the resting state, consistent with the idea of their cooperative interaction. Insights gained from EMS mutants highlight the critical roles played by CC and LRR domain of RXL, and all of the domains of Pm5e (including CC, NB‐ARC, LRR and IDR) in maintaining normal resistant function (Figure 1a, b). Therefore, we propose a working model for the RXL/Pm5e pair. In the pre‐activated state, RXL and Pm5e may form hetero‐complexes, which are maintained in the resting state through domain interactions. Upon *Bgt* invasion, Pm5e can recognize the effector either indirectly or directly, leading to the formation of a ternary complex containing RXL, Pm5e, and the powdery mildew effector. Interaction with the effector induces structural remodelling in Pm5e, leading to its activation. Simultaneously, RXL undergoes activation in a Pm5e‐activated‐dependent manner. This dependency arises from the fact that RXL lacks an independent NBD and HD1 domain, making it reliant on the activation initiated by Pm5e. The activation of RXL and Pm5e culminates in the formation of an activated RXL‐Pm5e resistosome, where Pm5e^NB‐ARC^ serves as the backbone, and the RXL^CC^ functions as the Ca^2+^ channel. Considering that a minimum of five independent functional CC domains is required to form a Ca^2+^ channel, we speculate that the RXL‐Pm5e resistosome may adopt a decameric structure, with RXL and Pm5e aligning in 1:1 stoichiometry in this complex. Within this complex, the five RXL^CC^ may overcome the repression imposed by Pm5e^CC^, and the proposed complex is inserted into the plasma membrane, and function as calcium‐permeable channels. This activation process could then trigger the initiation of the basal innate immune response (Figure S14).

The sensor NLR usually has an ID in well‐characterized NLRs. For example, RRS1 has the WRKY domain while RGA5 has the HMA domain. The NLR‐ID of a NLR pair accomplishes detection of effectors from pathogens, while its NLR partner is responsible for conversion of effector detection into defence activation. Integrated domains of NLR are though to function as decoys for pathogen effector targets (Maqbool *et al*., 2015). In RXL/Pm5e pair, Pm5e may act as sensor NLR. But, Pm5e has an IDR rather than ID. IDRs are abundant in eukaryotic proteomes and play a wide variety of essential roles. Rather than adopting a stable structure, IDRs exist in an ensemble of interconverting conformations, and thus, IDRs are ideally poised to act as sensors and actuators of cellular physicochemistry (Moses *et al*., 2023). The C‐terminal IDR with the C‐JID (C‐terminal jelly‐roll/Ig‐like) domain of WRR4, an NLR in *Arabidopsis*, plays a pivotal role in effector recognition (Castel *et al*., 2021). Likewise, the phosphorylation of the C‐terminal IDR of RRS1 is indispensable for PopP2 responsiveness (Guo *et al*., 2020). These findings underscore the vital importance of the IDR within sensor NLRs for effector recognition. In our previous study, we discovered that susceptible Pm5‐BMDM allele exhibits only one amino acid variation (I1011M) compared with Pm5e (Xie *et al*., 2020), and this amino acid is specifically located in the IDR. Furthermore, the EMS mutants M634, with a S1004L mutation in IDR, lose resistance to powdery mildew (Figure 1a,b). These findings suggest that the IDR may play a crucial role for the sensor NLR Pm5e in recognizing effectors. In the future, the cloning of the recognized effector of RXL/Pm5e and studying the role of the IDR in Pm5e for recognizing effectors holds great promise. Such investigations can offer valuable insights into the molecular mechanisms that govern disease resistance, contributing to a deeper understanding of the specific functions played by IDRs in NLRs upon effector recognition.

*RXL*/*Pm5e* pair has been shown not to negatively affect wheat growth and therefore has great potential for wheat breeding (Qiu *et al*., 2022). To effectively leverage the *RXL*/*Pm5e* pair in wheat breeding, we can use marker‐assisted selection to integrate this pair into elite breeding lines. Additionally, in elite cultivars carrying the none‐functional *Pm5e* allele (Xie *et al*., 2020), where the *RXL* allele is functional, we can employ genomic editing tools to precisely modify the susceptible *Pm5* allele into the resistant *Pm5e*. This strategy will expedite the development of resistant wheat varieties. Besides, there is growing evidence that engineering ID is effective to create new recognition specificity for NLR‐IDs and will become a practical approach for efficiently generating broad race spectrum resistance to multiple diseases (Cesari *et al*., 2022; Liu *et al*., 2021). The IDR within Pm5e appears pivotal in conferring powdery mildew resistance, functioning akin to the ID. Notably, this IDR is situated in the C‐terminus of the Pm5e and modifications in this region do not adversely impact the protein's structure. Hence, the targeted engineering of the Pm5e^IDR^ holds great potential to expand the molecular breeder's toolkit. This approach facilitates the development of customized *Pm5* variants designed to combat various powdery mildew isolates in wheat and related species.

## Methods

### Plant materials, wheat powdery mildew assay and infection type detection

The F_2:3_ population derived from crosses between resistant wheat cultivars Tangmai 4 (TM) or Fuzhuang 30 (FZ30) and their EMS susceptible mutants were used in the present study to analyse the relationship between susceptibility and mutation in the *RXL* or *Pm5e* using genetic and molecular techniques.

*B*. *graminis* f. sp. *tritici* isolate E20 was propagated and preserved in the susceptible bread wheat (*T*. *aestivum*) cultivar Chancellor or Xuezao (XZ). Detached leaves were placed in petri dishes containing 0.5% agar with 50 mg L^−1^ benzimidazole. Inoculation was carried out as described by Gong and colleagues (Zeng *et al*., 2014). The Petri dishes were incubated for 14 days in a greenhouse at 17 °C and 16‐h/8‐h photoperiod and infection types were scored as previously described (Xie *et al*., 2020).

### Transient protein expression and cell death assays in *N. benthamiana*

The constructs to express targeted proteins were transformed into *Agrobacterium* strain GV3101 (pMP90). *Agrobacterium* carrying relevant constructs were cultured in LB medium with appropriate antibiotics overnight at 28 °C with 200 rpm shaking. *Agrobacterium* cultures for relevant constructs were mixed to a final concentration OD_600_ = 0.5 and were harvested by centrifugation at 3000 ***g*** for 5 min. The sediment was resuspended in infiltration buffer (10 mm MES, pH5.6, 10 mm MgCl_2_ and 200 μM acetosyringone) and incubated in at room temperature for ~3 h to induce virulence. The mixed suspensions were infiltrated to 4–5‐week‐old *N*. *benthamiana* with a needleless 1‐mL syringe. Hypersensitive response (HR) was visually inspected 7 days after agroinfiltration.

### Split‐luciferase assays

The split‐luciferase assays were performed to investigate the interactions between RXL and Pm5e using constructs pICSL86977OD‐Flag‐nLUC and pICSL86977OD‐Flag‐cLUC. Corresponding sequences of *RXL* and *Pm5e* were amplified using respective primers and inserted into vectors using Golden Gate methods (Lin *et al*., 2022). The fusion constructs were transformed into *Agrobacterium* strain GV3101 (pMP90) and transiently expressed in *N*. *benthamiana*. After about 48 h, luciferin (1 mm) was spread evenly on the abaxial side of leaf to activate luciferase, and the leaves were treated in the dark for 10 min and were detected for imaging (NightOWL II LB 983 In Vivo Imaging System with WinLight Software; BERTHOLD TECHNOLOGIES, Germany). Three leaves were utilized for each experiment, and three independent biological repeats were conducted. Western blots with anti‐ FLAG‐HRP antibodies were executed for detecting the presence of the recombinant protein.

### Yeast two‐hybrid assays

To validate the interaction between the CC domains of RXL and Pm5e, we cloned their CC domain coding sequences into the vectors pGBKT7 and pGADT7 using specific primers to generate respective constructs (Table S5). The yeast strain Y2H Gold was used for transformation following the Matchmaker Gold Yeast Two‐Hybrid System manual (Clontech). The resulting transformants were spotted on SD (−Trp/−Leu) for growth and then spotted on SD (−Trp/−Leu/–His) selection medium. The growth observed on the selection plates indicates protein interaction. Plates were incubated at 30 °C for 72 h and yeast growth was observed.

### Construction of vectors for gene editing and silencing

The CRISPR direct website (http://crispr.dbcls.jp/) was used to design two sgRNAs and an intermediate vector pMETaU6.1 was used as the template to be amplified by the primers E3‐RXL‐F and E3‐RXL‐R, containing two sgRNAs, respectively (Table S5). The amplification products were digested by *Bsa*I (NEB) and inserted into the linearized vector pLGYE‐3, which is under the TaU3 promoter (Li *et al*., 2021). The resulting construct pLGYE3‐RXL was transformed into Fielder/TM F_1_ plants to generate the RXL‐edited plants with homozygous *Pm5e*. Primers (RXL‐Edit‐MD) were applied to check the positive plants. Additionally, primers (RXL‐AF and WGGB202) designed to amplify the target mutations of *RXL* and detect the presence of *Pm5e* (Table S5).

To generate the *RXL* RNAi constructs, a unique 183‐bp CDS fragment of *RXL* was amplified using specific primers and inserted as inverted repeats into the RNAi vector pLGY‐02 to generate hairpin RNAi construct. The *RXL* RNAi plasmid was introduced into Fielder/TM F_1_ plants via *Agrobacterium* (EHA105)‐mediated transformation. All primer sequences used for cloning can be found in Table S5.

### BSMV‐VIGS

The BSMV‐mediated gene silencing system was used to silence *RXL* and *Pm5e*, respectively, as previously described with some modification (Yuan *et al*., 2011). We cloned the initial 210 bp sequences of *RXL* and *Pm5e* from their start codons into pCa‐γbLIC in antisense direction to generate constructs pCaBS‐γ:RXL and pCaBS‐γ:Pm5e (Table S5). All constructs were verified by Sanger sequencing. The resulting constructs were transformed into *Agrobacterium* strain GV3101 and equimolar amount of cultures containing pCaBS‐α, pCaBS‐β and pCaBS‐γ:RXL or pCaBS‐γ:Pm5e were expressed transiently into 4–5‐week‐old *N. benthamiana* plants, using the empty vector (pCa‐γbLIC) as control. Two‐leaf stage leaves of resistant wheat cultivar TM were inoculated artificially with extract from the infiltrated *N. benthamiana* leaves. Post‐inoculation plants were covered with black plastic sheet for 24 h to keep moist and maintained in incubator with day/night temperatures of 22/18 °C and a 16‐/8‐h photoperiod. At 14 days after virus infection, the third leaves were infected with the avirulent isolate *Bgt* E20. After 10 days, powdery mildew phenotypes were identified and stained with 0.6% (w/v) Coomassie blue solution to observe the hypha as the method previously reported (Xie *et al*., 2020). Furthermore, a part of infected leaf pieces was used for further gene silencing expression analyses for detection of *RXL* or *Pm5e* expression with qRT‐PCR.

### Protein sequence analysis and structural modelling

The prediction of protein modelling and interactions of RXL and Pm5e was performed using AlphaFold2 on a high‐performance computer at The Sainsbury Laboratory (Evans *et al*., 2022; Jumper *et al*., 2021). For each protein sequence, the highest ranked result was selected for further analysis. Protein structures were visualized and aligned using ChimeraX (Pettersen *et al*., 2021).

### Protein extraction and co‐immunoprecipitation

*Agrobacterium*‐infiltrated leaves of 0.2 g were harvested at 3 dpi and promptly flash‐frozen in liquid nitrogen within 2‐mL tubes. After freezing, the samples were ground to a fine powder in liquid nitrogen. Next, 400 μL of extraction buffer (50 mm Tris, 50 mm NaCl, 5 mm MgCl_2_, 10% glycerol, 10 mm DTT, 1× plant protease inhibitor cocktail) was added to each sample, followed by gentle vertexing on ice until complete homogenization. The homogenized samples were then centrifuged at 13 000 rpm for 10 min at 4 °C, and the resulting lysate was carefully transferred to fresh 2‐mL tubes. This lysate was subjected to a second centrifugation step at 13000 rpm for 5 min at 4 °C to obtain a clarified lysate for each sample. Extracts were boiled for 5 min and then centrifuged at 13 000 rpm for 1 min at 4 °C and supernatants were collected with SDS loading buffer for protein gel blot analysis.

The co‐immunoprecipitation (co‐IP) procedure was performed as described previously (Lin *et al*., 2022). For protein extraction from *N. benthamiana*, fresh leaves were ground in liquid nitrogen and homogenized in the IP extraction buffer (50 mm Tris–HCl, pH 7.5, 150 mm NaCl, 1 mm EDTA, 10% glycerol, 1% Triton X‐100, 1 mm PMSF and 1× protease inhibitor cocktail). Different combinations of supernatants were incubated with anti‐Flag beads for 2 h at 4 °C and then washed four times with extraction buffer. The affinity beads were boiled for 5 min in SDS loading buffer to elute the bound proteins. The immunoprecipitated proteins were analysed by western blot. Antibodies used for co‐IP and western blot are described below: Flag (1:10 000), HA (1:5000), GFP (1:2000), V5 (1:2000).

### BN‐page

*Agrobacterium*‐infiltrated leaves were collected at 3 dpi, and the cleared lysate was obtained as described above. For BN‐PAGE analysis, an equivalent volume of soluble supernatant from each sample, was transferred to new tubes and diluted according to the manufacturer's instructions by adding Native PAGE 5% G‐250 sample, 4× Sample Buffer (Invitrogen), and water. Subsequently, these samples were promptly loaded onto Native PAGE 3–12% Bis‐Tris gels, alongside NativeMark unstained protein standard (Invitrogen), to estimate the size of the identified protein species. Electrophoresis was carried out at 150 V in dark buffer for 50 min, followed by 250 V in light buffer for 1 h. The proteins were then transferred onto polyvinylidene difluoride (PVDF) membranes and fixed by incubating with 8% acetic acid for 15 min. After washing the membranes three times with water, they were air‐dried in a fume hood for 30 min. Ethanol was utilized to reactivate the membranes for proper visualization of the unstained native protein marker. Following labeling of the native protein marker on the membranes and several subsequent washes with methanol and water, the membranes were immunoblotted using Image Quant 800 (Cytiva).

### Evolutionary analyses of RXL and Pm5e orthologous proteins

To decipher the origin and evolutionary history of RXL and Pm5e orthologs, we surveyed 122 representative plant species, including 19 non‐flowering and 103 flowering taxa, with a particular focus on 36 Poales species (Table S2). We used an integrated approach of similarity searching and phylogenetic analysis to identify RXL and Pm5e orthologs. For RXL orthologs identification, a PSI‐BLAST search was executed against 122 plant proteomes with the RXL amino acid sequence from *T. aestivum* (accession No.: TraesCS7B03G1192600) as the query and an *e*‐value threshold of 10^−5^ (Altschul *et al*., 1997). Full‐length amino acid sequences of significant hits were aligned with representative NLRs using the localpair algorithm implemented in MAFFT (Katoh and Standley, 2013). Large‐scale phylogenetic analyses were performed using the approximate maximum likelihood method implemented in FastTree (Price *et al*., 2010). Sequences clustering with TraesCS7B03G1192600 were extracted and annotated using HMMSCAN in HMMER 3.3.2 (http://hmmer.org/) with an *e*‐value threshold of 0.01. Proteins lacking the NB‐ARC domain and forming a monophyletic group were regarded as RXL orthologous proteins. For Pm5e orthologs identification, we used the NB‐ARC domain sequence of Pm5e from *T. aestivum* (accession No.: QHN12679) as the query to search against 122 plant proteomes using PSI‐BLAST with *e*‐value cut‐off value of 10^−5^ (Altschul *et al*., 1997). Significant hits were extracted and aligned with NB‐ARC domains of representative NLRs using HMMALIGN in HMMER 3.3.2 (http://hmmer.org/). Phylogenetic analyses were performed using the approximate maximum likelihood method implemented in FastTree (Price *et al*., 2010). Orthologous sequences that formed a cluster with QHN12679 were identified as Pm5e orthologous proteins. We also used the tBLASTn algorithm as a supplementary method to search against plant genomes. Domain architectures of RXL and Pm5e orthologous proteins were annotated using HMMSCAN in HMMER 3.3.2 (http://hmmer.org/).

To elucidate the phylogenetic relationships among RXL and Pm5e orthologous proteins, full‐length RXL proteins and closely related NLR proteins were aligned using MAFFT with the localpair strategy (Katoh and Standley, 2013). The NB‐ARC domain of Pm5e orthologous proteins along with closely related NLR proteins were aligned using the same strategy. Alignments were refined with TrimAL and manually edited for enhanced accuracy (Capella‐Gutiérrez *et al*., 2009). Phylogenetic analyses were performed using the maximum likelihood method implemented in IQ‐TREE 2 (Minh *et al*., 2020). The best‐fit substitution model was determined by the ModelFinder algorithm (Kalyaanamoorthy *et al*., 2017). Node support values were estimated through the UFBoot approach (Hoang *et al*., 2017). Phylogenetic trees were annotated and visualized using iTOL (Letunic and Bork, 2021). Outgroups were designated based on the aforementioned large‐scale phylogenetic trees.

### Gene pair exploration

To trace back to when the RXL and Pm5e orthologs formed the head‐to‐head pair configuration, we selected 13 representative plant species covering the diversity of Poales (Figure S13). All the NLRs of these 13 plants were aligned using HMMALIGN in HMMER 3.3.2, followed by large‐scale phylogenetic analyses with FastTree (Price *et al*., 2010). Proteins closely related to RXL and Pm5e clades were retrieved for subsequent analysis. Full‐length sequences of these representative proteins were aligned together with full‐length RXL and Pm5e proteins through localpair strategy in MAFFT (Katoh and Standley, 2013) and the alignment was refined using TrimAL (Capella‐Gutiérrez *et al*., 2009). Phylogenetic analysis was performed using FastTree (Price *et al*., 2010) and the outgroup proteins were selected based on the large‐scale phylogenetic trees aforementioned. Adjacent genomic pairs of proteins were identified, labelled as pairs, and linked graphically using iTOL (Letunic and Bork, 2021).

## Funding

This work was supported by grants from the National Key Research and Development Program of China (2022YFF1001503, 2023YFD1200402), the National Natural Science Foundation of China (U21A20224), the Major Project of Agricultural Biological Breeding (2023ZD0402502, 2023ZD0407001.08) and Youth Innovation Promotion Association CAS (2021093). This project was also supported by grants from the Hainan Seed Industry Laboratory (B21HJ0111), Key Research and Development Program of Zhejiang (2024SSYS0099), and Key Research and Development Program of Hebei (22326305D). G.G. was supported by the special project of long‐term overseas training for young scientific and technological talents in 2022 of CAS.

## Author contributions

Z.L., G.G. T.H and J.J. conceived and designed the research. G.G., K.B. and Y.H. participated in the material preparation and molecular experiments with helpful insights from W.S., M.X., L.Y., D.Y., Y.Z., Y.C. and H.L. Z.G. and G.H. performed bioinformatics analyses. G.G., K.B., Y.H., H.Z., Q.W., P.L., M.L., L.D., J.X., Y.C., P.Z., K.Z., B.L., W.L., L.D., Y.Y., D.Q. and C.Y. participated in the field experiment. G.G., Z.L., G.H., T.H and J.J. wrote the manuscript. All authors edited the manuscript.

## Competing interests

The authors declare no competing interests.

## Supporting information


**Figure S1** The expression patterns of *RXL* and *Pm5e* upon *Bgt* inoculation. Samples from the treatments were collected at 0, 4, 6, 12, 24, 48 and 72 h post inoculation (hpi) with *Bgt* E20, with each time point consisting of three biological replicates. Transcript levels were examined using qRT‐PCR. *TaACTIN* was used as an internal control. Error bars represent the standard error of the means (SEMs) of three independent experiments.
**Figure S2** Mutations in *RXL* or *Pm5e* co‐segregated with *Bgt* infection type in respective F_2:3_ genetic families. The genomic region covering *RXL* and *Pm5e* in wild types was illustrated with the green rectangle, while the corresponding region in mutants were depicted with the yellow rectangle. Genetic families with homozygous wild types exhibited homozygous resistant (HR), those with homozygous mutant types showed homozygous susceptibility (HS) and those with heterozygous types displayed segregation (Seg). The numbers in parentheses next to the nucleotides indicated number of families.
**Figure S3** Confirmation of functional identity of *RXL* and *Pm5e* by BSMV‐VIGS. (a, b) Schematic diagram of *RXL* (a) and *Pm5e* (b). Yellow and orange bars indicate regions selected as BSMV‐VIGS targets, while black bars below the diagrams denote regions targeted for qRT‐PCR amplification to detect expression. (c, d) Expression levels of the *RXL* (c) and *Pm5e* (d) of BSMV:γ, BSMV:*RXL* and BSMV:*Pm5e*. The expression was assessed by qRT‐PCR. Statistical analysis was done using a two‐tailed Student's *t*‐test at *P* < 0.05 on the basis of three biological replicates. Error bars, mean ± SEM. (e) Representative macroscopic and microscopic images showing the results of the BSMV‐VIGS experiment. BSMV:γ denote control with an empty silencing construct. BSMV:*RXL* and BSMV:*Pm5e* denote silencing constructs that target *RXL* and *Pm5e*, respectively. Scale bar, 100 μm.
**Figure S4** The relative expression of *RXL* and *Pm5e* in *RXL*‐edited mutants and relative expression of *Pm5e* in *RXL*‐RNAi plants. (a) The relative expression of *RXL* and *Pm5e* in five dependent *RXL*‐edited mutants, with non‐edited lines used as the control. (b) The relative expression of *Pm5e* in two dependent *RXL*‐RNAi lines, with TM used as the control. The expression was evaluated through qRT–PCR, and statistical analysis was performed using a two‐tailed Student's *t*‐test with significance set at *P* < 0.05, based on three biological replicates. Error bars represent the mean ± SEM.
**Figure S5** The *RXL* allele in Fielder is functional. (a) Gene structure of *RXL* and *Pm5* alleles. (b, c) Protein sequence alignment of RXL and RXL‐Fielder (b) and protein sequence alignment of Pm5e and Pm5‐Fielder (c). Differences were marked with different colours. (d) The relative expression levels of *RXL‐Fielder* or *Pm5e* in BSMV:γ, BSMV:*RXL‐Fielder* and BSMV:*Pm5e* were assessed using qRT‐PCR. The BSMV‐VIGS assay was conducted in *Pm5e* overexpression (Pm5e‐OE) transgenic plants. BSMV:*RXL‐Fielder* represents the silencing construct targeting *RXL‐Fielder*, while BSMV:γ serves as a negative control with an empty silencing construct, and BSMV:*Pm5e* indicates the silencing constructs targeting *Pm5e*, used as a positive control. Statistical analysis was performed with a two‐tailed Student's *t*‐test at *P* < 0.05 based on three biological replicates, with error bars representing mean ± SEM. (e) Representative macroscopic images demonstrate the results of the BSMV‐VIGS experiment. (f) The phenotype observed upon *Bgt* inoculation was documented in the resistant parent (FZ30), the susceptible Fielder used for genetic transformation, and T_1_ transgenic plants containing *RXL* with the native promoter (T_1__RXL_Com), *Pm5e* with the native promoter (T_1__Pm5e_Com), and *Pm5e* with the maize ubiquitin promoter (T_1__Pm5e_OE). Additionally, a schematic diagram illustrating the genetic background was included.
**Figure S6** The domain analysis of RXL and Pm5e. The predicted domains of RXL (a) and Pm5e (b) are represented. Conserved motifs characteristic of the NB‐ARC domain are highlighted in blue characters, and the corresponding names of motifs are indicated with red letters.
**Figure S7** Protein domain architecture of Pm5e and prediction of intrinsically disordered regions (IDRs). IDRs were predicted using the online tool PONDR (www.pondr.com).
**Figure S8** Prediction of RXL and Pm5e hetero‐ and homo‐dimer. View of predicted RXL‐Pm5e heterodimer (a), RXL homodimer (b), and Pm5e homodimer (c), generated using AlphaFold2. Scores (0.2 pTM + 0.8 ipTM) range from 0 (worst) to 1 (best). The schematic of domains of RXL and Pm5e are shown above.
**Figure S9** Negative control of bimolecular fluorescent complementation (BiFC) and the protein expression of fusion proteins in BiFC assays. (a), No fluorescence signal was observed when co‐expressing RXL or Pm5e with control vectors. Scale bars, 50 mm. (b), Proteins were extracted 3 days after the infiltration and analysed through SDS‐PAGE, followed by immunoblotting with anti‐Flag antibodies (α‐Flag).
**Figure S10** The protein expression of fusion proteins in split‐luciferase assays. Proteins were extracted 3 days after the infiltration and analysed suing SDS‐PAGE. Subsequently, immunoblotting was performed with anti‐Flag antibodies (α‐Flag).
**Figure S11** MHD motif mutants of RXL and Pm5e are unable to induce HR. (a) Alignment of a segment of the ARC2 domain sequences of Rx, MLA10, RGA4, RGA5, RXL and Pm5e containing the region containing the MHD motif (red box). (b) The wild‐type and MHD mutant constructs of RXL and Pm5e, including RXL‐GFP, RXL_PTV_‐GFP, Pm5e‐GFP, Pm5e_VHV_‐GFP, Pm5e_VVH_‐GFP, were expressed in *N. benthamiana* and analysed for cell death induction. (c) RXL‐GFP in combination with Pm5e_VHV_‐GFP and Pm5e_VVH_‐GFP, as well as Pm5e‐GFP in combination with RXL_PTV_‐GFP, were co‐infiltrated in *N. benthamiana*. Cell death was visualized 7 days after infiltration. The same results were obtained in at least three independent experiments. (d) Proteins were extracted 3 days after the infiltration of wild‐type and MHD mutant constructs of RXL and Pm5e and analysed by immunoblotting with anti‐GFP antibodies (α‐GFP).
**Figure S12** Phylogenetic relationship of RXL, Pm5e orthologous proteins and related NLRs. (a) Phylogenetic relationship among Pm5e orthologs and representative NLRs. The phylogeny was reconstructed based on the NB‐ARC domain. The Pm5e orthologous proteins are highlighted in blue background. The number near the Pm5e clade node is the UFBoot support value. Tips are represented by solid circles with different colours according to their host taxonomy. (b) Phylogenetic relationship of RXL orthologs and related CNLs. The phylogeny was reconstructed based on the full‐length RXL orthologs and related NLRs. The RXL clade is highlighted in green and numbers near nodes represent UFBoot support values. Tips are represented by solid circles with different colours according to their host taxonomy. The domain architecture is shown next to the protein.
**Figure S13** Pair connection of partial NLRs from 13 representative plant species. NLRs closely related to Pm5e and RXL were retrieved from the large‐scale phylogeny. The phylogeny was reconstructed based on full‐length sequences using an approximate maximum likelihood method. Yellow curved lines connect adjacent NLRs, while the lines linking Pm5e‐related proteins and RXL‐related proteins are emphasized in darker shade.
**Figure S14** The possible working model for RXL/Pm5e pair. In the absence of pathogens, RXL and Pm5e undergo hetero‐oligomerization. When recognizing the effector, Pm5e, with an intact NB‐ARC domain, undergoes a structural change to form a decamer with RXL, causing de‐repression of Pm5e^CC^ towards RXL^CC^. This allows RXL^CC^ to assemble and serve as a Ca^2+^ channel, triggering downstream immune responses.

**Table S1** Responses of wild types, mutants and F_2:3_ families to the *Bgt* isolate E20.
**Table S2** The information of 122 species used for identification of RXL and Pm5e orthologs proteins analyses.
**Table S3** Domain architectures of Pm5e orthologous proteins identified in plant proteomes.
**Table S4** Domain architectures of RXL orthologous proteins identified in plant proteomes.
**Table S5** Primers used in this study.

## Data Availability

All data and materials needed to repeat the work are available. Alignments and phylogenetic trees generated in this study have been deposited to Mendeley Data and are available at https://data.mendeley.com/preview/993mmzvspv?a=9cea24ad‐d245‐4adf‐998e‐1741c3d81028.
