# Supplementary material for: The wheat NLR pair RXL/Pm5e confers resistance to powdery mildew
Source: Plant Biotechnol J. 2025 Jan 22;23(4):1260–76. doi: 10.1111/pbi.14584 (PMC11933841; doi:10.1111/pbi.14584)
Supplement: Supplementary file 1 — Figure S1 The expression patterns of RXL and Pm5e upon Bgt inoculation. Samples from the treatments were collected at 0, 4, 6, 12, 24, 48 and 72 h post inoculation (hpi) with Bgt E20, with each time point consisting of three biological replicates. Transcript levels were examined using qRT‐PCR. TaACTIN was used as an internal control. Error bars represent the standard error of the means (SEMs) of three independent experiments. Figure S2 Mutations in RXL or Pm5e co‐segregated with Bgt infection type in respective F2:3 genetic families. The genomic region covering RXL and Pm5e in wild types was illustrated with the green rectangle, while the corresponding region in mutants were depicted with the yellow rectangle. Genetic families with homozygous wild types exhibited homozygous resistant (HR), those with homozygous mutant types showed homozygous susceptibility (HS) and those with heterozygous types displayed segregation (Seg). The numbers in parentheses next to the nucleotides indicated number of families. Figure S3 Confirmation of functional identity of RXL and Pm5e by BSMV‐VIGS. (a, b) Schematic diagram of RXL (a) and Pm5e (b). Yellow and orange bars indicate regions selected as BSMV‐VIGS targets, while black bars below the diagrams denote regions targeted for qRT‐PCR amplification to detect expression. (c, d) Expression levels of the RXL (c) and Pm5e (d) of BSMV:γ, BSMV:RXL and BSMV:Pm5e. The expression was assessed by qRT‐PCR. Statistical analysis was done using a two‐tailed Student's t‐test at P < 0.05 on the basis of three biological replicates. Error bars, mean ± SEM. (e) Representative macroscopic and microscopic images showing the results of the BSMV‐VIGS experiment. BSMV:γ denote control with an empty silencing construct. BSMV:RXL and BSMV:Pm5e denote silencing constructs that target RXL and Pm5e, respectively. Scale bar, 100 μm. Figure S4 The relative expression of RXL and Pm5e in RXL‐edited mutants and relative expression of Pm5e in RXL‐RNAi plants. (a) The [file PBI-23-1260-s001.docx]

0.30

0.25

0.20

Relative expression

0.15

0.10

*RXL*

*Pm5e*

0.05

0.00

0 4 6 12 24 48 72

hpi

**Fig. S1.** The expression patterns of *RXL* and *Pm5e* upon *Bgt* inoculation. Samples from the treatments were collected at 0, 4, 6, 12, 24, 48, and 72 hours post-inoculation (hpi) with *Bgt* E20, with each time point consisting of three biological replicates. Transcript levels were examined using qRT-PCR. *TaACTIN* was used as an internal control. Error bars represent the standard error of the means (SEMs) of three independent experiments.

M3274/FZ30 M3289/FZ30 M1650/FZ30 M608/TM M1006/FZ30

*RXL*

*Pm5e*

WT

Hybrid

Mutant

IT HR

Seg

HS

RXL.c70 G/G (36)

G/A (52)

A/A (31)

RXL.c431 G/G (30)

G/A (60)

A/A (24)

RXL.c1525 C/C (17)

C/T (32)

T/T (10)

Pm5e.c1701 G/G (12)

G/A (26)

A/A (10)

Pm5e.c2181 G/G (12)

G/A (27)

A/A (9)

**Fig. S2.** Mutations in *RXL* or *Pm5e* co-segregated with *Bgt* infection type in respective F_2_:3 genetic families. The genomic region covering *RXL* and *Pm5e* in wild types was illustrated with the green rectangle, while the corresponding region in mutants were

depicted with the yellow rectangle. Genetic families with homozygous wild types exhibited homozygous resistant (HR), those with homozygous mutant types showed homozygous susceptibility (HS), and those with heterozygous types displayed segregation (Seg). The numbers in parentheses next to the nucleotides indicated number of families.

**A B**

*RXL*

*Pm5e*

**C**

1.2

1

0.8

*RXL/Actin*

0.6

0.4

0.2

0

**BSMV:EV**

**D**

1.4

*****

BSMV:ψ

BSMV:*RXL*

1.2

1

*Pm5e/Actin*

0.8

0.6

0.4

0.2

0

**BSMV:RXL**

*

BSMV:ψ BSMV:*Pm5e*

**BSMV:EV**

**BSMV:Pm5e**

**E**

BSMV:ψ

BSMV:*RXL*

BSMV:*Pm5e*


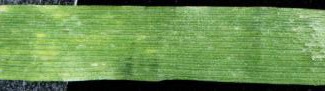

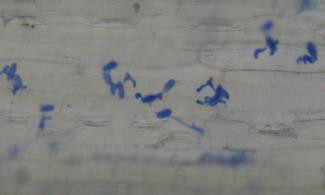

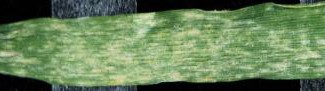

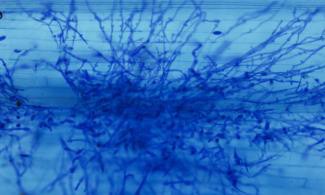

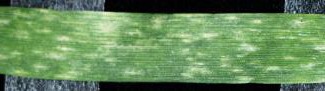

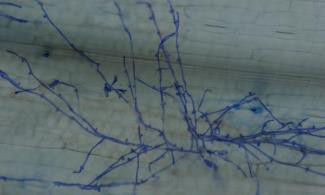


**Fig. S3.** Confirmation of functional identity of *RXL* and *Pm5e* by BSMV-VIGS. (A, B) Schematic diagram of *RXL* (A) and *Pm5e* (B). Yellow and orange bars indicate regions selected as BSMV-VIGS targets, while black bars below the diagrams denote regions targeted for qRT-PCR amplification to detect expression. (C, D) Expression levels of the *RXL* (C) and *Pm5e* (D) of BSMV:γ, BSMV:*RXL,* and BSMV:*Pm5e.* The expression was assessed by qRT-PCR. Statistical analysis was done using a two-tailed Student’s t-test at *P* < 0.05 on the basis of three biological replicates. Error bars, mean ± SEM. (E) Representative macroscopic and microscopic images showing the results of the BSMV- VIGS experiment. BSMV:ψ denote control with an empty silencing construct. BSMV:*RXL* and BSMV:*Pm5e* denote silencing constructs that target *RXL* and *Pm5e*, respectively. Scale bar, 100 µm.

A

ns

0.60

ns

0.50

0.40

Relative expression

0.30

0.20

RXL-nonED RXL-ED1 RXL-ED2 RXL-ED3 RXL-ED4 RXL-ED5

0.10

0.00

*RXL*

*Pm5e*

B

1.5

ns

ns

1.0

*Pm5e*/*TaActin*

0.5

0.0

**Fig. S4.** The relative expression of *RXL* and *Pm5e* in *RXL*-edited mutants and relative expression of *Pm5e* in *RXL*-RNAi plants. (A) The relative expression of *RXL* and *Pm5e* in five dependent *RXL*-edited mutants, with non-edited lines used as the control. (B) The relative expression of *Pm5e* in two dependent *RXL*-RNAi lines, with TM used as the control. The expression was evaluated through qRT–PCR, and statistical analysis was performed using a two-tailed Student’s t-test with significance set at *P* < 0.05, based on three biological replicates. Error bars represent the mean ± SEM.

**A**

TGA

*RXL Pm5e*

ATG ATG TAA

1 kb

TGA

*RXL-Fielder Pm5-Fielder*

ATG ATG TAA

Retrotransposon (RLC_Taes_Ada_3BctgD-82)


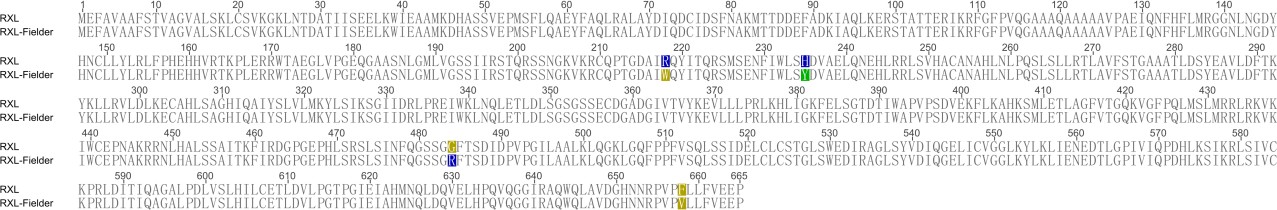
**B**


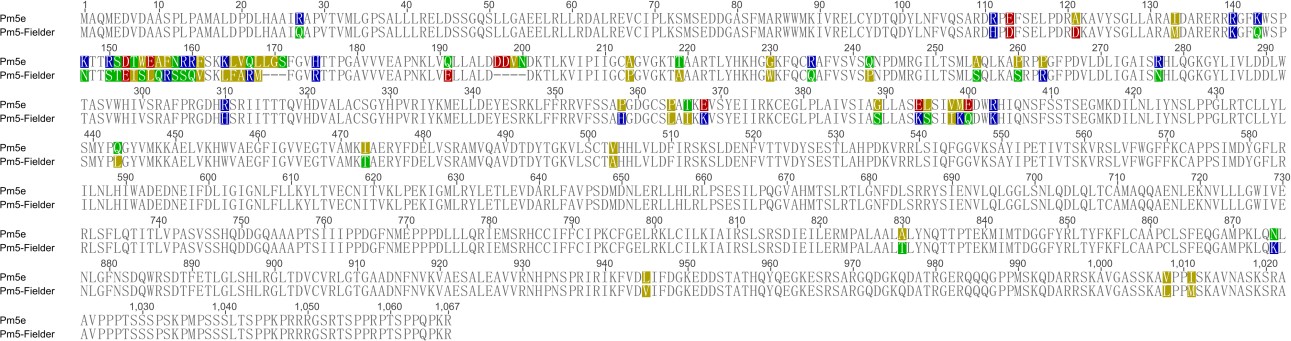
**C**

**D**

1.5

✱✱✱

1.0

*Pm5e*/*Actin*

1.5

1.0

✱✱

*RXL-Fielder*/*Actin*

**F**


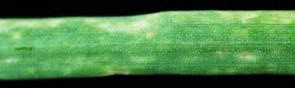
FZ30

Resistant


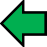


*RX*d*L Pm5e*

0.5

0.5

Fielder

Susceptible


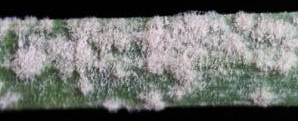
*RXL-Fielder Pm5-Fielder*


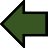

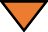


0.0

0.0

T_1__RXL_Com Susceptible

*RXL-Fielder Pm5-Fielder*


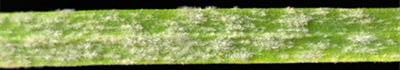


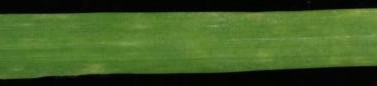
**E**


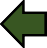

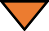

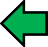


*RXL*

BSMV:ψ


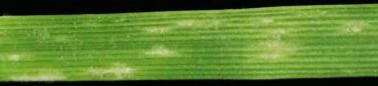
BSMV:*Pm5e*

T_1__Pm5e_Com


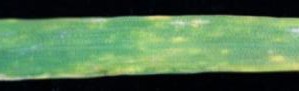

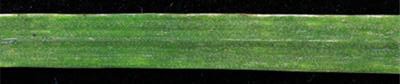
T_1__Pm5e_OE

Resistant

Resistant

*RXL-Fielder Pm5-Fielder*


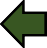

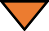


*Pm5e*

*RXL-Fielder Pm5-Fielder*


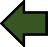

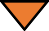


*Pm5e*


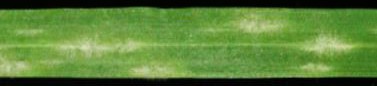
BSMV:*RXL-Fielder*

Retrotransposon

**Fig. S5.** The *RXL* allele in Fielder is functional. (A) Gene structure of *RXL* and *Pm5* alleles. (B, C) Protein sequence alignment of RXL and RXL- Fielder (B) and protein sequence alignment of Pm5e and Pm5-Fielder (C). Differences were marked with different colors. (D) The relative expression levels of *RXL-Fielder* or *Pm5e* in BSMV:γ, BSMV:*RXL-Fielder* and BSMV:*Pm5e* were assessed using qRT-PCR. The BSMV-VIGS assay was conducted in *Pm5e* overexpression (Pm5e-OE) transgenic plants. BSMV:*RXL-Fielder* represents the silencing construct targeting *RXL- Fielder*, while BSMV:γ serves as a negative control with an empty silencing construct, and BSMV:*Pm5e* indicates the silencing constructs targeting *Pm5e*, used as a positive control. Statistical analysis was performed with a two-tailed Student’s t-test at P < 0.05 based on three biological replicates, with error bars representing mean ± SEM. (E) Representative macroscopic images demonstrate the results of the BSMV-VIGS experiment.

(F) The phenotype observed upon *Bgt* inoculation was documented in the resistant parent (FZ30), the susceptible Fielder used for genetic transformation, and T_1_ transgenic plants containing *RXL* with the native promoter (T_1__RXL_Com), *Pm5e* with the native promoter (T_1__Pm5e_Com), and *Pm5e* with the maize ubiquitin promoter (T_1__Pm5e_OE). Additionally, a schematic diagram illustrating the genetic background was included.

**A B**

**RXL Pm5e**

**MEFAVAAFSTVAGVALSKLCSVKGKLNTDATIISEELKWIE**

**CC AAMKDHASSVEPMSFLQAEYFAQLRALAYDIQDCIDSFNA**

**MAQMEDVDAASPLPAMALDPDLHAAIRAPVTVMLGPSALLLRELDSS**

**atypical GQSLLGAEELRLLRDALREVCIPLKSMSEDDGASFMARWWMKIVRE**

**Broken NB-ARC**

**KMTTDDEFADKI AQLKERSTATTERIKRFGF**

**PVQGAAAQAAAAAVPAEIQNFHFLMRGGNLN**

**RNB S-D**

**GDYHNCLLYLRLFPHEHHVRTKPLERR WTAEGLVPGEQGAASNLGMLVGSSIIRSTQRSSNGKVKRC W IAEGF**

**QPTGDAIRQYITQRSMSENFIWLSHDVAELQNEH**

**M HD**

**CC LCYDTQDYLNFVQSARDRPEFSELPDRAKAVYSGLLARAIDARERRRG**

**FKWSPKTT**

**RSDTWEAFNRRFSKKLVQLLGSFGVHTTPGAVV**

**P-loop VEAPNKLVQLLALDDDVNDKTLKVIPIIGCAGVGKTTAARTLYHKHG GKFQCRAFVSVSQNPDMRGILTSMLAQLKAPRPPGFPDVLDLIGAISR**

**RNB S-A**

**HLQGKGYLIVLDDLWTASVWHIVSRAFPRGDHRSRIITTTQVHDVALA**

**K inase 2 RNB S-B**

**LRRLSVHACANAHLNLPQS**

**NB-ARC CSGYHPVRIYKMELLDEYESRKLFFRRVFSSAPGDGCSPATKEVSYEII**

**LSLLRTLAVFSTGAAATLDSYEAVLDFTKYKL**

**GLPL**

**RNB S-C**

**LRVLDLKECAHLSAGHIQAIYSLV**

**LMKYLSIKSGIIDRLPREIWKLNQ**

**RKCEGLPLAIVSIAGLLASELSIVMEDWRHIQNSFSSTSEGMKDILNLI YNSLPPGLRTCLLYLSMYPQGYVMKKAELVKHWVAEGFIGVVEGTVA**

**LETLDLSGSGSSECDGADGIVTVYKEVLLLPR**

**RNB S-D**

**W IAEGF**

**LRR**

**LKHLIGKFELSGTDTIWAPVPSDVEKFLKAHKSM LETLAGFVTGQKVGFPQLMSLMRR LRKVKIWCEPNAKRRNLHALSSAITKFIRDGPGEPH LSRSLSINFQGSSGGFTSDIDPVPGI LAALKLQGKLGQFPPFVSQ LSSIDELCLCSTGLSWEDIRAGLSYVDIQGELICVGG LKYLKLIENEDTLGPIVIQPDHLKS IKRLSIVCKPRLDITIQAGALPD LVSLHILCETLDVLPGTPGIEIAHMNQ LDQVELHPQVQGGIRAQWQLAVDGHNNRPVPFLLFVEEP**

**LRR**

**IDR**

**MKIAERYFDELVSRAMVQAVDTDYTGKVLSCTVHH LVLDFIRSKSLD**

**M HD ENFVTTVDYSESTLAHPDK VRRLSIQFGGVKSAYIPETIVTSK VRSLVFWGFFKCAPPSIMDYGF LRILNLHIWADEDNEIFDLIGIGNLFL LKYLTVECNITVKLPEKIGMLRY LETLEVDARLFAVPSDMDNLER LLHLRLPSESILPQGVAHMTS LRTLGNFDLSRRYSIENVLQLGGLSN LQDLQLTCAMAQQAENLEKNVLLLGWIVERLSF LQTITLVPASVSSHQDDGQAAAPTSIIIPPDGFNMEPPPDLL LQRIEMSRHCCIFFCIPKCFGELRK LCILKIAIRSLSRSDIEILERMPA LAALALYNQTTPTEKMIMTDGGFYR LTYFKFLCAAPCLSFEQGAMPK LQNLNLGFNSDQWRSDTFETLGLSHLRG LTDVCVRLGTGAADNFNVKVAESALEAVVRNHPN SPRIRIKFVDLI**

**FDGKEDDSTATHQYQEGKESRSARGQDGKQDATRGERQQQGPPMSK QDARRSKAVGASSKAVPPISKAVNASKSRAAVPPPTSSSPSKPMPSSSLT SPPKPRRRGSRTSPPRPTSPPQPKR**

**Fig. S6.** The domain analysis of RXL and Pm5e. The predicted domains of RXL (A) and Pm5e (B) are represented. Conserved motifs characteristic of the NB-ARC domain are highlighted in blue characters, and the corresponding names of motifs are indicated with red letters.

**Pm5e (Average Prediction Score: 0.3221)**

| **CC** |  | **NB-ARC** | **LRR** | **IDR** |
| --- | --- | --- | --- | --- |
|  |  |  |  |  |


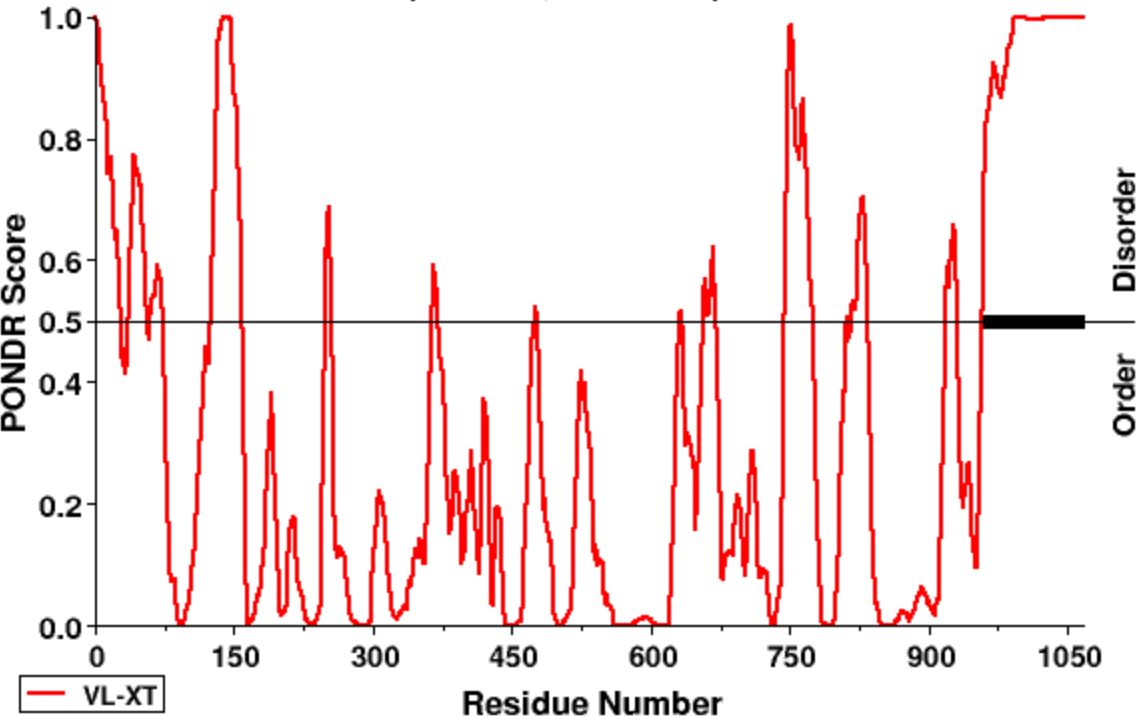


**Fig. S7.** Protein domain architecture of Pm5e and prediction of intrinsically disordered regions (IDRs)**.** IDRs were predicted using the online tool PONDR (www.pondr.com).

**A**

| CC | WHD | LRR |
| --- | --- | --- |

**RXL**


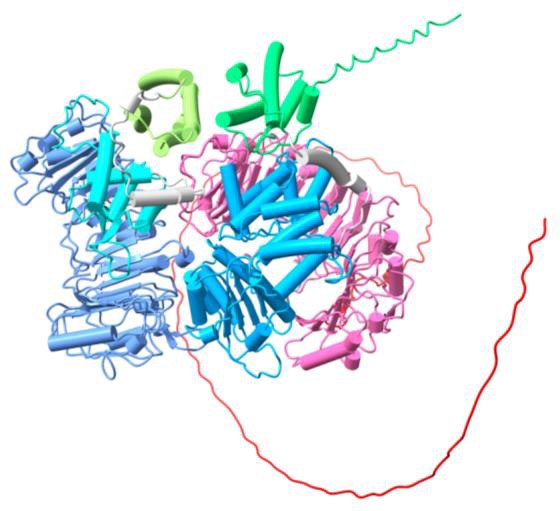
**Pm5e**

| CC | NB-ARC | LRR | IDR |
| --- | --- | --- | --- |

**B**

RXL

| CC | WHD | LRR |
| --- | --- | --- |


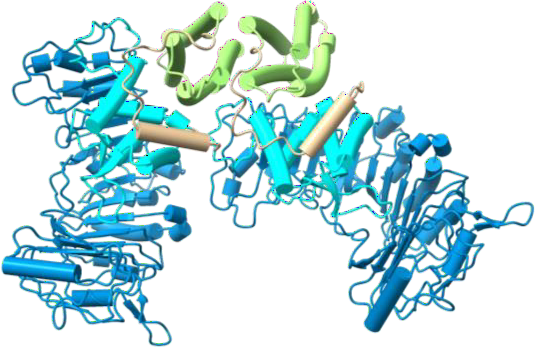


**C**

Pm5e

| CC | NB-ARC | LRR | IDR |
| --- | --- | --- | --- |

**Score = 0.86**

**Score = 0.43**


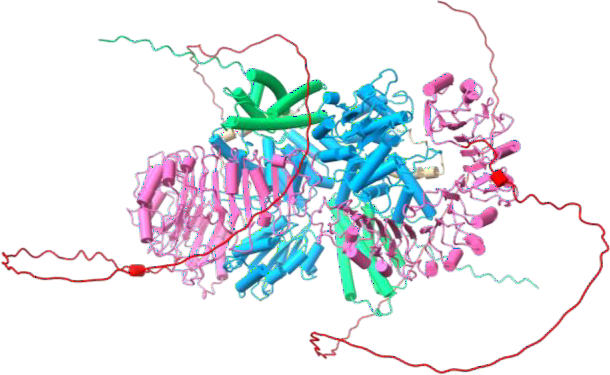
**Score = 0.63**

**Fig. S8.** Prediction of RXL and Pm5e hetero- and homo-dimer. View of predicted RXL- Pm5e heterodimer (A), RXL homodimer (B), and Pm5e homodimer (C), generated using Alphafold2. Scores (0.2 pTM + 0.8 ipTM) range from 0 (worst) to 1 (best). The schematic of domains of RXL and Pm5e are shown above.

**A**


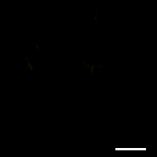

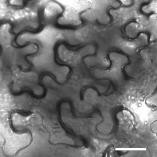

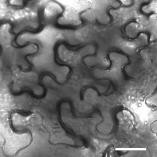


| **B** |  | | | | | | |
| --- | --- | --- | --- | --- | --- | --- | --- |
| RXL-nYFP | **+** | **-** | **-** | **-** | **-** | **-** |  |
| RXL-cYFP | **-** | **+** | **-** | **-** | **-** | **-** |  |
| Pm5e-nYFP | **-** | **-** | **+** | **-** | **-** | **-** |  |
| Pm5e-cYFP | **-** | **-** | **-** | **+** | **-** | **-** |  |
| Pm1a-nYFP | **-** | **-** | **-** | **-** | **+** | **-** |  |
| Pm1a-cYFP | **-** | **-** | **-** | **-** | **-** | **+** |  |
| α-Flag |  |  |  |  |  |  | 170 kDa  130 kDa |
|  |  |  |  |  |  |  | 100 kDa |
| Ponceau |  |  |  |  |  |  |  |

RXL-nYFP+Pm1a-cYFP


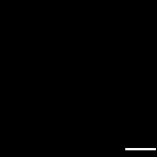

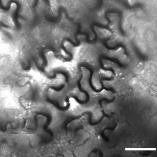

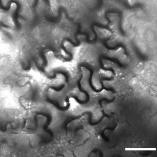

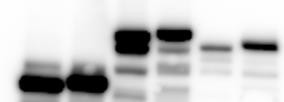

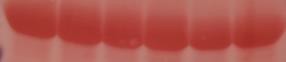


RXL-cYFP+Pm1a-nYFP


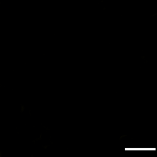

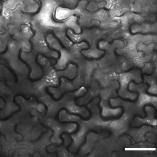

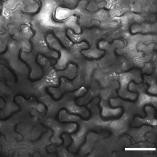


Pm5e-nYFP+Pm1a-cYFP


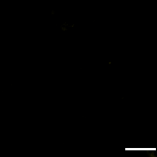

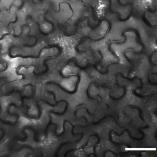

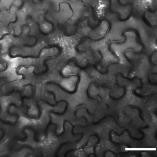


Pm5e-cYFP+Pm1a-nYFP

**Fig. S9.** Negative control of bimolecular fluorescent complementation (BiFC) and the protein expression of fusion proteins in BiFC assays. (A), No fluorescence signal was observed when co-expressing RXL or Pm5e with control vectors. Scale bars, 50 mm. (B), Proteins were extracted 3 days after the infiltration and analyzed through SDS- PAGE, followed by immunoblotting with anti-Flag antibodies (α-Flag).

RXL-nLUC **+ - - - - -**

RXL-cLUC **-**

Pm5e-nLUC **-**

Pm5e-cLUC **-**

**+ - - - -**

**- + - - -**

**- - + - -**

Pm1a-nLUC **- - - - + -**

Pm1a-cLUC **+**

α-Flag

170 kDa

130 kDa


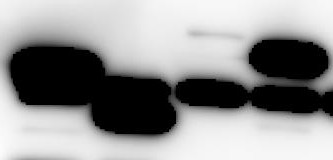

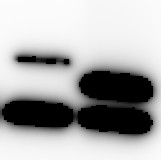


100 kDa

Ponceau


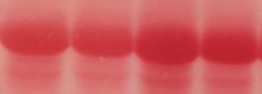

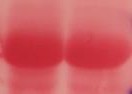


**Fig. S10.** The protein expression of fusion proteins in split-luciferase assays. Proteins were extracted 3 days after the infiltration and analyzed suing SDS-PAGE. Subsequently, immunoblotting was performed with anti-Flag antibodies (α-Flag).

**A**

**RX**

**MLA10 RGA4 RXL**

**Pm5e**

**RGA5**

**MHD motif**

**B C D**


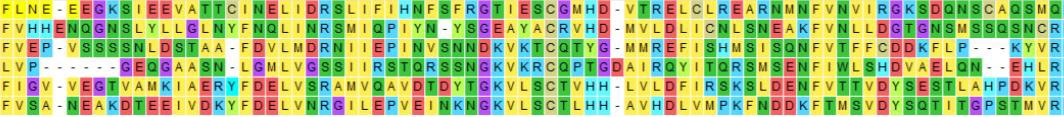


RXL-GFP **+ - - - -**


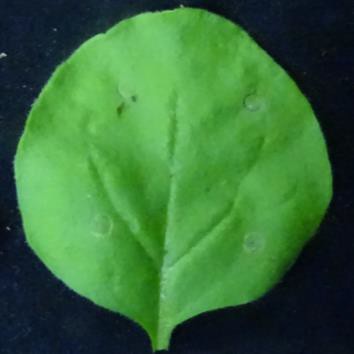


**RXL-GFP Pm5eVVH-GFP**

**RXL-GFP**

**Pm5eVHV-GFP**

**RXLPTV-GFP Pm5e-GFP**

**RXL-GFP Pm5e-GFP**


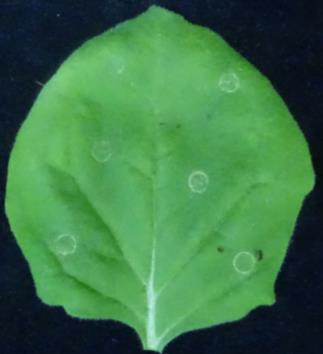


**RXL-GFP**

**Pm5e-GFP**

**Pm5eVHV-GFP**

**RXLPTV-GFP**

**RXL-GFP Pm5e-GFP**

**Pm5eVVH-GFP**

RXLPTV-GFP

**- + - - -**

Pm5e-GFP **- - + - -**

Pm5eVVH

-GFP

**- - - + -**

Pm5eVHV-GFP

**- - - - +**

α-GFP

130 kDa

100 kDa


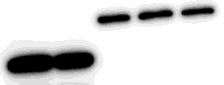


Ponceau


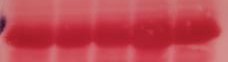


**Fig. S11.** MHD motif mutants of RXL and Pm5e are unable to induce HR. (A) Alignment of a segment of the ARC2 domain sequences of Rx, MLA10, RGA4, RGA5, RXL and Pm5e containing the region containing the MHD motif (red box). (B) The wild-type and MHD mutant constructs of RXL and Pm5e, including RXL-GFP, RXLPTV-

GFP, Pm5e-GFP, Pm5eVHV-GFP, Pm5eVVH-GFP, were expressed in *N. benthamiana* and

analyzed for cell death induction. (C) RXL-GFP in combination with Pm5eVHV-GFP and Pm5eVVH-GFP, as well as Pm5e-GFP in combination with RXLPTV-GFP, were co-

infiltrated in *N. benthamiana*. Cell death was visualized 7 days after infiltration. The same results were obtained in at least three independent experiments. (D) Proteins were extracted 3 days after the infiltration of wild-type and MHD mutant constructs of RXL and Pm5e and analyzed by immunoblotting with anti-GFP antibodies (α-GFP).


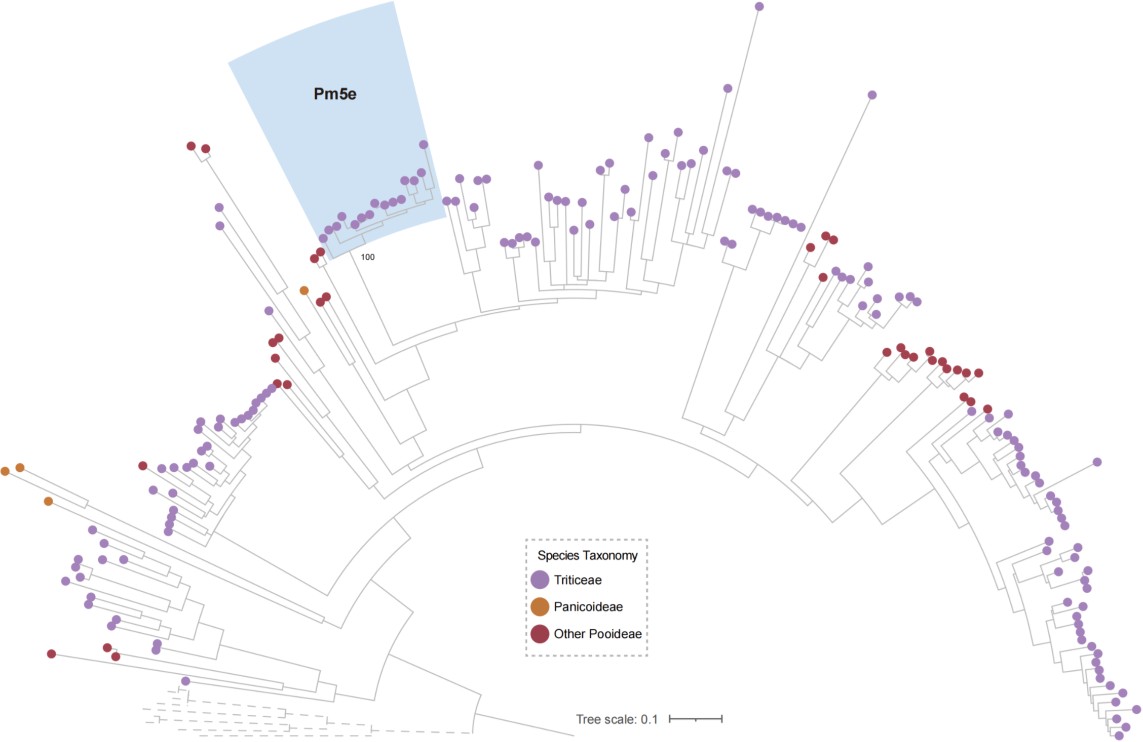
**A**

**B**


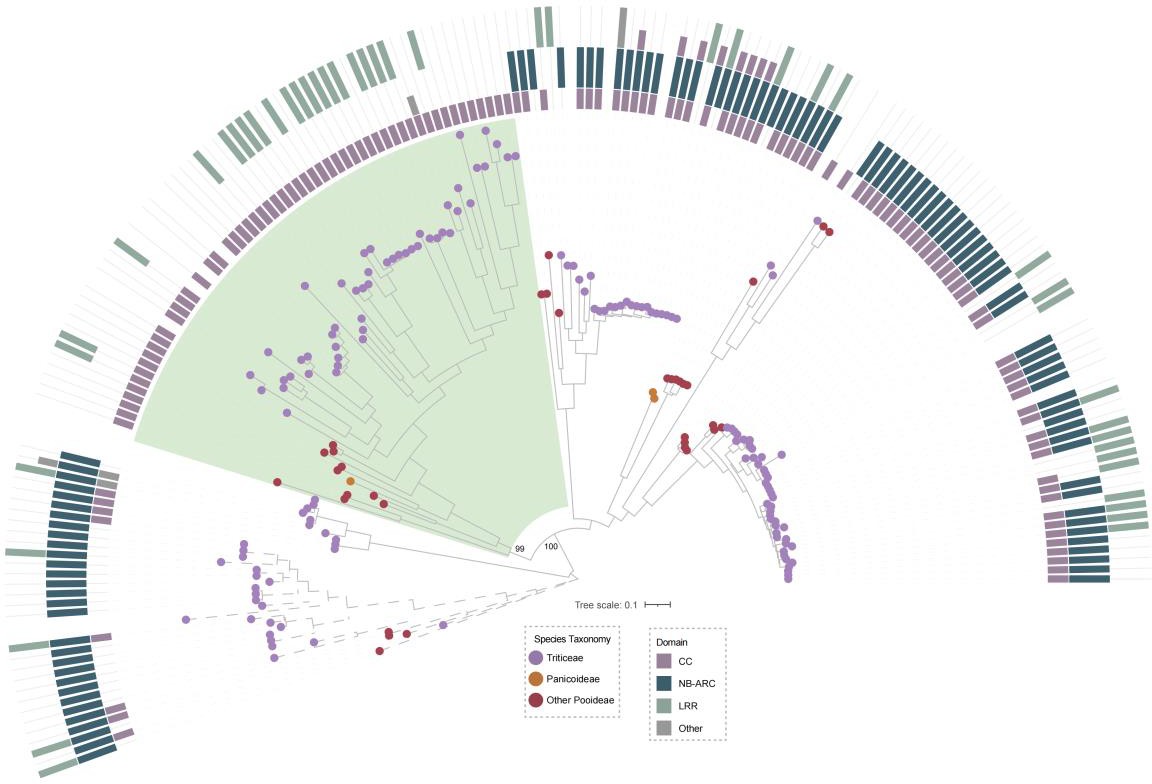


**RXL**

**Fig. S12.** Phylogenetic relationship of RXL, Pm5e orthologous proteins and related NLRs. (A) Phylogenetic relationship among Pm5e orthologs and representative NLRs. The phylogeny was reconstructed based on the NB-ARC domain. The Pm5e orthologous proteins are highlighted in blue background. The number near the Pm5e clade node is the UFBoot support value. Tips are represented by solid circles with different colors according to their host taxonomy. (B) Phylogenetic relationship of RXL orthologs and related CNLs. The phylogeny was reconstructed based on the full-length RXL orthologs and related NLRs. The RXL clade is highlighted in green and numbers near nodes represent UFBoot support values. Tips are represented by solid circles with different colors according to their host taxonomy. The domain architecture is shown next to the protein.


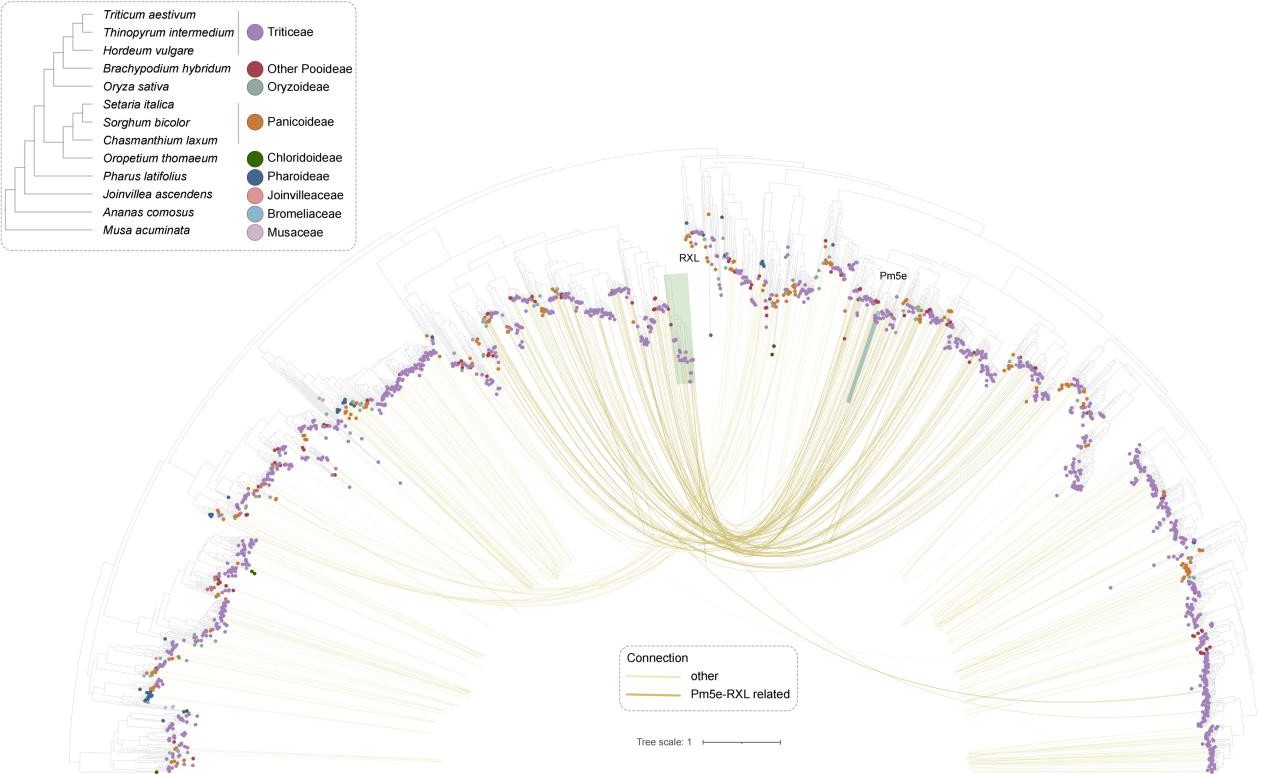


**Fig. S13.** Pair connection of partial NLRs from 13 representative plant species. NLRs closely related to Pm5e and RXL were retrieved from the large-scale phylogeny. The phylogeny was reconstructed based on full-length sequences using an approximate maximum likelihood method. Yellow curved lines connect adjacent NLRs, while the lines linking Pm5e-related proteins and RXL-related proteins are emphasized in darker shade.


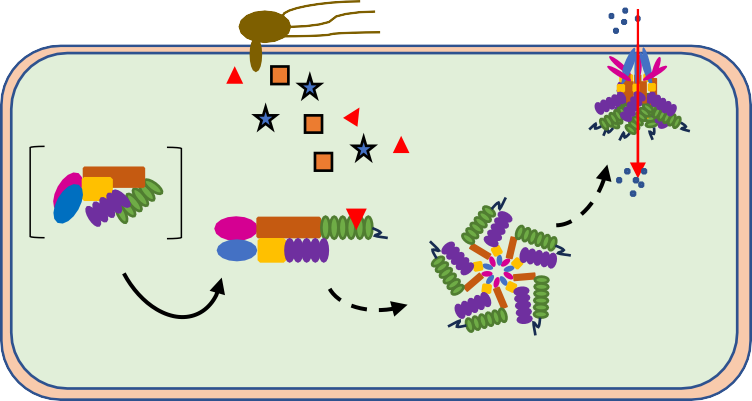


**Ca2+**

Pm5e

RXL

x N Pm5e ***** effector RXL

**Fig. S14.** The possible working model for RXL/Pm5e pair. In the absence of pathogens, RXL and Pm5e undergo hetero-oligomerization. When recognizing the effector, Pm5e, with an intact NB-ARC domain, undergoes a structural change to form a decamer with RXL, causing de-repression of Pm5eCC towards RXLCC. This allows RXLCC to assemble and serve as a Ca2+ channel, triggering downstream immune responses.
